# hnRNP A1/A2 and Sam68 collaborate with SRSF10 to control the alternative splicing response to oxaliplatin-mediated DNA damage

**DOI:** 10.1038/s41598-018-20360-x

**Published:** 2018-02-02

**Authors:** Alexandre Cloutier, Lulzim Shkreta, Johanne Toutant, Mathieu Durand, Philippe Thibault, Benoit Chabot

**Affiliations:** 1 Department of Microbiology and Infectious Diseases, Faculty of Medicine and Health Sciences, Université de Sherbrooke, Sherbrooke, Quebec J1E 4K8 Canada; 2 RNomics Platform of the Université de Sherbrooke, Université de Sherbrooke, Sherbrooke, Quebec J1E 4K8 Canada

## Abstract

Little is known about how RNA binding proteins cooperate to control splicing, and how stress pathways reconfigure these assemblies to alter splice site selection. We have shown previously that SRSF10 plays an important role in the *Bcl-x* splicing response to DNA damage elicited by oxaliplatin in 293 cells. Here, RNA affinity assays using a portion of the *Bcl-x* transcript required for this response led to the recovery of the SRSF10-interacting protein 14-3-3ε and the Sam68-interacting protein hnRNP A1. Although SRSF10, 14-3-3ε, hnRNP A1/A2 and Sam68 do not make major contributions to the regulation of *Bcl-x* splicing under normal growth conditions, upon DNA damage they become important to activate the 5′ splice site of pro-apoptotic Bcl-xS. Our results indicate that DNA damage reconfigures the binding and activity of several regulatory RNA binding proteins on the *Bcl-x* pre-mRNA. Moreover, SRSF10, hnRNP A1/A2 and Sam68 collaborate to drive the DNA damage-induced splicing response of several transcripts that produce components implicated in apoptosis, cell-cycle control and DNA repair. Our study reveals how the circuitry of splicing factors is rewired to produce partnerships that coordinate alternative splicing across processes crucial for cell fate.

## Introduction

Alternative splicing generates proteomic diversity that drives biological complexity. Splicing decisions are regulated by RNA binding proteins (RBPs) that interact with splicing enhancer and silencer elements. Although these elements are usually located close to regulated splice sites^1^, they can also be found at some distance and brought in closer physical proximity by RNA looping^2^. Specific RBPs can display enhancer or silencer activity depending on where they bind on a pre-mRNA and may collaborate, synergize or antagonize with neighboring RBPs^1^. Documenting the combinatorial contribution of several RBPs in the regulation of a specific splicing decision remains an understudied question in splicing control, and only a few model systems have been used to explore this complexity (e.g. *Bcl-x*, *c-src, CD45* and *Fas*^3–6^). Another emerging but still poorly understood area concerns how the interactions between regulatory RBPs are restructured following physiological cues and environmental stresses such as DNA damage. On this theme, recent studies have identified co-transcriptional splicing events that are disrupted by DNA damage (reviewed in refs^7,8^). For example, different types of DNA damaging agents impact the co-transcriptional splicing activity of EWS on transcripts encoding apoptosis and DNA repair components^9,10^. DNA damage also favors the assembly of a complex between BRCA1 and splicing factors at DNA repair genes that stimulates splicing^11^.

We have selected the *Bcl-x* gene (aka *BCL2L1*) to study how multiple RBPs converge to regulate a simple splicing decision that involves two competing 5′ splice site (5′ss) producing the pro-apoptotic Bcl-xS and the anti-apoptotic Bcl-xL splice variants. *Bcl-x* also provides an ideal system to study how DNA damage remodels splicing regulation to encourage the production of a pro-apoptotic splice variant. A large number of RBPs have been implicated in the homeostatic regulation of *Bcl-x* splicing^3^: these include the SR proteins SRSF1^12–15^, SRSF2^16^, SRSF3^17^, SRSF7^17^, SRSF9^13^ and SRSF10^18^, the hnRNP proteins A1^12^, PTBP1^17^, K^19^ and F/H^20,21^, the RNA binding proteins Sam68^12^, SF3B1^22^, RBM4^23^, RBM11^24^, RBM25^25^, RBM10^26^, TRA2β^17^, the exon-junction components RNPS1, Acinus, SAP18 and eIF4A3/Y14^27^, as well as the transcription factors TCERG1^28^ and FBI-1^28,29^. In addition, several kinases and signalling pathways converge to regulate *Bcl-x* splicing, including the tyrosine kinase FYN1 that phosphorylates Sam68^12^, the aurora kinase A that affects SRSF1 phosphorylation^14^, the phosphatase 2A inhibitor protein SET that interacts with hnRNP K to stimulate its repressor activity^30^, the PP1 phosphatase that targets SF3B1 and SRSF10^22,31^, and more than a dozen other kinases and phosphatases for which we know little about their target RBPs^14,32–34^.

One interesting feature uncovered about homeostatic *Bcl-x* splicing regulation is the role of PKC in enforcing repression of the 5′ss of Bcl-xS, and the loss of this repression following activation of the DNA damage response (DDR) using platinium agents^33,35^. Notably, the impact of both PKC inhibition and oxaliplatin on *Bcl-x* splicing require the SB1 element, a 361 nt-long ill-defined region located approximately 150 nt upstream of the 5′ss of Bcl-xS. The only regulatory factors that have been associated with this region are RNPS1, whose repressive activity on the Bcl-xS 5′ss requires a small 10 nt-long region in the central portion of SB1^27^, and TCERG1, which relieves a transcription pausing site in the downstream portion of the element important for repression of the Bcl-xS 5′ss^28^.

To gain further insight into the contribution of SB1 to *Bcl-x* splicing control, we carried out a mutational analysis of the SB1 element, and used affinity chromatography to recover the 14-3-3ε and the hnRNP A1/A2 proteins. 14-3-3ε is known to interact with SRSF10^31^, while hnRNP A1/A2 collaborates with Sam68 to control *Bcl-x* splicing^12^. Interestingly, we find that the contributions of 14-3-3ε, hnRNP A1/A2 and Sam68 are most apparent following DNA damage when they become important to activate the 5′ss of pro-apoptotic Bcl-xS. By extending our analysis to other transcripts associated with the DDR, we report that the combinatorial contribution of hnRNP A1/A2, Sam68 and SRSF10 to alternative splicing of DDR-relevant transcripts is broadly reconfigured after DNA damage, allowing coordinated splicing decisions to control the production of components that orchestrate the cellular response to DNA damage.

## Results

### Dissecting the SB1 element

**Figure 1:**
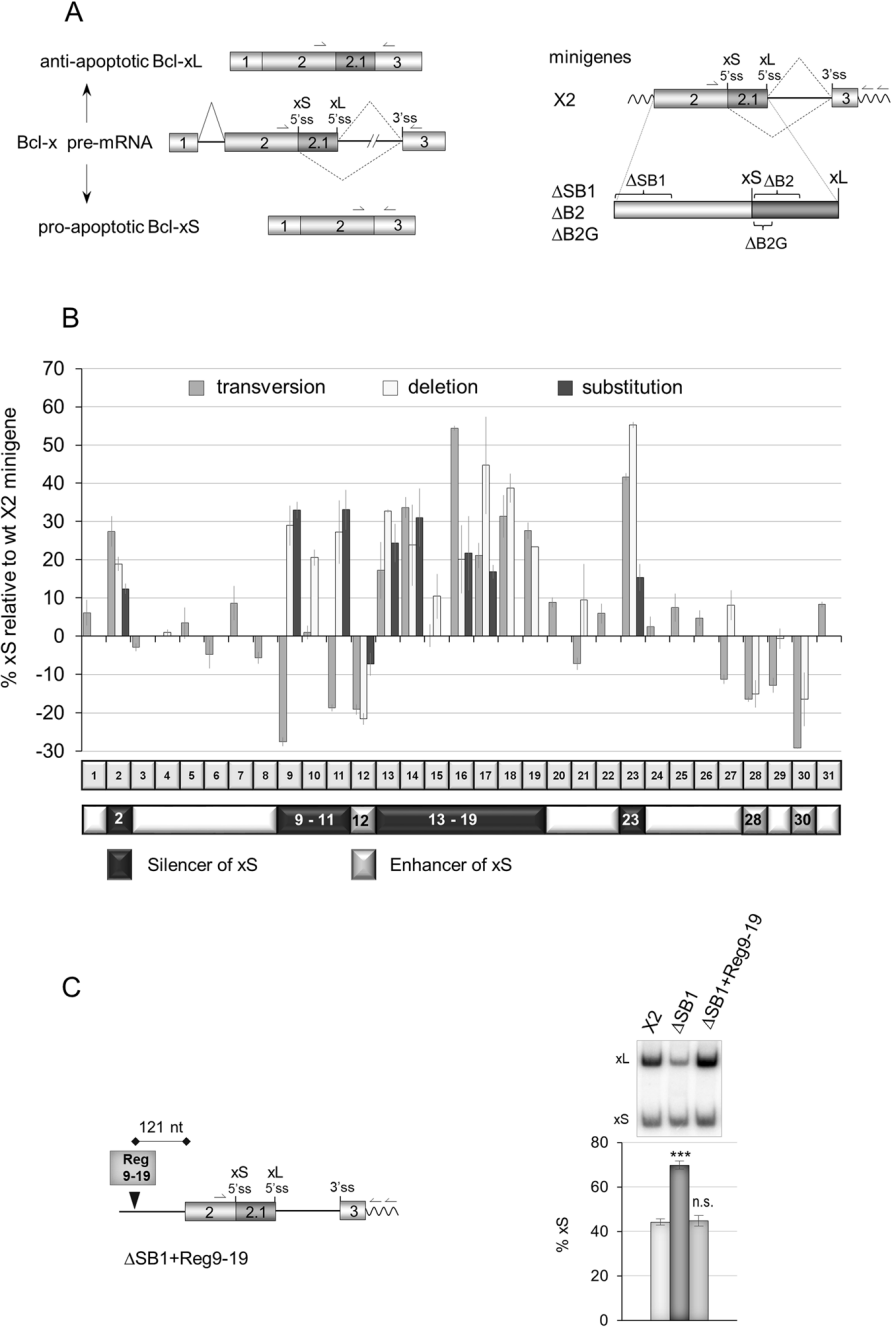
Mutagenesis of the SB1 element in *Bcl-x* exon 2. (**A**) Structure of the human *Bcl-x* pre-mRNA and its two splice variants anti-apoptotic Bcl-xL and the shorter pro-apoptotic isoform Bcl-xS. The portion included in minigene X2 and deleted in minigenes ΔSB1, ∆B2 and ∆B2G is shown on the right. Primer positions for RT-PCR assays from endogenous and minigene *Bcl-x* transcripts are shown. (**B**) The graph plots the percentage of Bcl-xS mRNA obtained by RT-PCR analysis for each mutation relative to the wild-type X2 minigene. The SB1 element was divided into 31 segments of 10 nt. One round of mutations changed A, G, C and T into T, C, G and A, respectively (transversions). A second set of mutations produced deletions of selected segments. Finally, specific segments were mutated by changing the wild-type sequence for GACTCAGTGT. Categorization or segments as displaying enhancer or silencer activity only considers the impact of deletions greater than 10 percentage points. (**C**) RT-PCR assays on total RNA extracted from ECR293 cells transfected with X2, ∆SB1 and ∆SB1 + Reg9-19 minigenes. The Reg9-19 region was inserted 279 nucleotides upstream of Bcl-xS 5′ss. The percentage of Bcl-xS is an average of triplicates. Error bars indicate SD. Asterisks represent significant *P* values (two-tailed Student’s *t* test) comparing the means between samples and their respective controls. *P < 0.05, **P < 0.01 and ***P < 0.001; n.s. = not significant.

The 361-nucleotide (nt)-long element SB1 begins approximately 150 nt upstream of the Bcl-xS 5′ss (Fig. 1A) and behaves globally as a silencer for this 5′ss^33^. We have shown previously that a 10-nt portion of SB1 (dubbed region 18 or Reg18) is required for RNPS1 to act as a repressor^27^. We also identified a RNA polymerase II (RNAPII) pausing site within SB1 (Reg23) that represses the use of the Bcl-xS, and is relieved by the elongation and splicing-related factor TCERG1^28^. To investigate in more details the contribution of other portions of SB1, we produced an extensive set of substitutions mutants that we tested in ECR293 cells. Essentially, a transversion mutant was made for each consecutive 10-nt segment along the length of a 310-nt portion of SB1, producing 31 subregions (Fig. 1B, Supplementary Fig. S1). For several transversion mutants displaying an effect, we tested the impact of a simple 10-nt deletion mutant and of another 10-nt randomly selected substitution mutant carrying the sequence GACTCAGTGT.

Fifteen of the 31 transversion mutants identified non-contiguous regulatory regions (Fig. 1B). Testing deletion mutants for each of the 10-nt segment confirmed several regulatory regions, identified a new one (Reg15) and yielded two mutants shifting in the direction opposite than what was observed with the first set of substitutions (Reg9 and 11). The different impact of transversion and deletion mutants may be expected since each transversion creates a new sequence with some that may affect splicing. In all cases tested, the GACTCAGTGT substitution mutants confirmed the impact of the deletion mutants. Using a threshold for splicing change of 10 percentage points with the deletion mutants, we present a refined splicing control map for SB1 with a central 110 nt-long silencer region (Reg9-19) interrupted by a weak enhancer (Reg12) (Fig. 1B). This core silencer is flanked on either side by two silencer elements (Reg2 and Reg23), and two downstream enhancer elements (Reg28 and Reg30). Inserting Reg9-19 in the ΔSB1 minigene at a site that respects its natural distance to the Bcl-xS 5′ss imposed repression of the Bcl-xS 5′ss (Fig. 1C).

### 14-3-3ε and hnRNP A1/A2 associate with SB1

To identify factors that associate with the SB1 element, we employed two chromatographic strategies. First, we stably co-expressed in 293 cells the MS2-TAP protein and the SB1 element tagged with 10 repeats of the MS2 binding site (Supplementary Fig. S2A). A mutated version of this element (SB1∆3) that harbors deletions of segments Reg11, Reg17 and Reg23 was used as a control. Individually or in combination, these deletions abrogate the SB1-mediated splicing repression (Supplementary Fig. S2B). Total RNA from stable ECR293 clones was tested by RT-PCR for expression of SB1-MS2 or SB1∆3-MS2 (Supplementary Fig. S2C). Expression of the MS2-TAP was confirmed by immunoblotting (Supplementary Fig. S2D). Relying on the MS2-TAP protein, we then carried out tandem affinity purification to capture transcripts containing MS2 binding sites and bound proteins. Pilot experiments indicated that the MS2 protein and SB1 transcripts were efficiently recovered by this procedure. A silver-stained gel displaying recovered proteins revealed a band at about 29 kDa in the SB1-MS2 but not in SB1∆3-MS2-derived complexes (Supplementary Fig. S2E). The band was cut out and analyzed by liquid chromatography-mass spectrometry (LC-MS/MS). Mascot-based analysis of acquired LC-MS/MS data revealed the peptide KEAAENSLUAYKA as a partial sequence (amino acids 142–154) of the 14-3-3ε protein (confidence level: peptide ≥95% and protein ≥99%).

In parallel, we conducted RNA affinity chromatography using SB1 transcripts and HeLa nuclear extracts (Supplementary Fig. S2F). In this case, we used the central repressive region and its immediate neighboring subregions, the 130 nt-long Reg8–20. As a control for binding specificity, we used a 154 nt-long transcript made of multiple cloning sites from pCDNA3.1+. The RNA was covalently linked to adipic acid dihydrazide agarose beads to which was added the extract. Proteins were then eluted with increasing concentrations of NaCl. The 200 nM NaCl eluate revealed a silver-stained band of about 35–36 kDa appearing with the Reg8–20 RNA but not with the control transcript (Supplementary Fig. S2G). The band was cut out and analyzed by mass spectrometry to reveal multiple peptides belonging to various members of the hnRNP A/B family of proteins, with the most extensive protein coverage obtained with the hnRNP A1 and A2 proteins, respectively at 41% and 49%. The presence of four putative binding sites for hnRNP A1 and A2 within the target sequence (Supplementary Fig. S2H) supports the binding of these proteins to SB1.

### 14-3-3ε contributes to the *Bcl-x* splicing response to DNA damage

**Figure 2:**
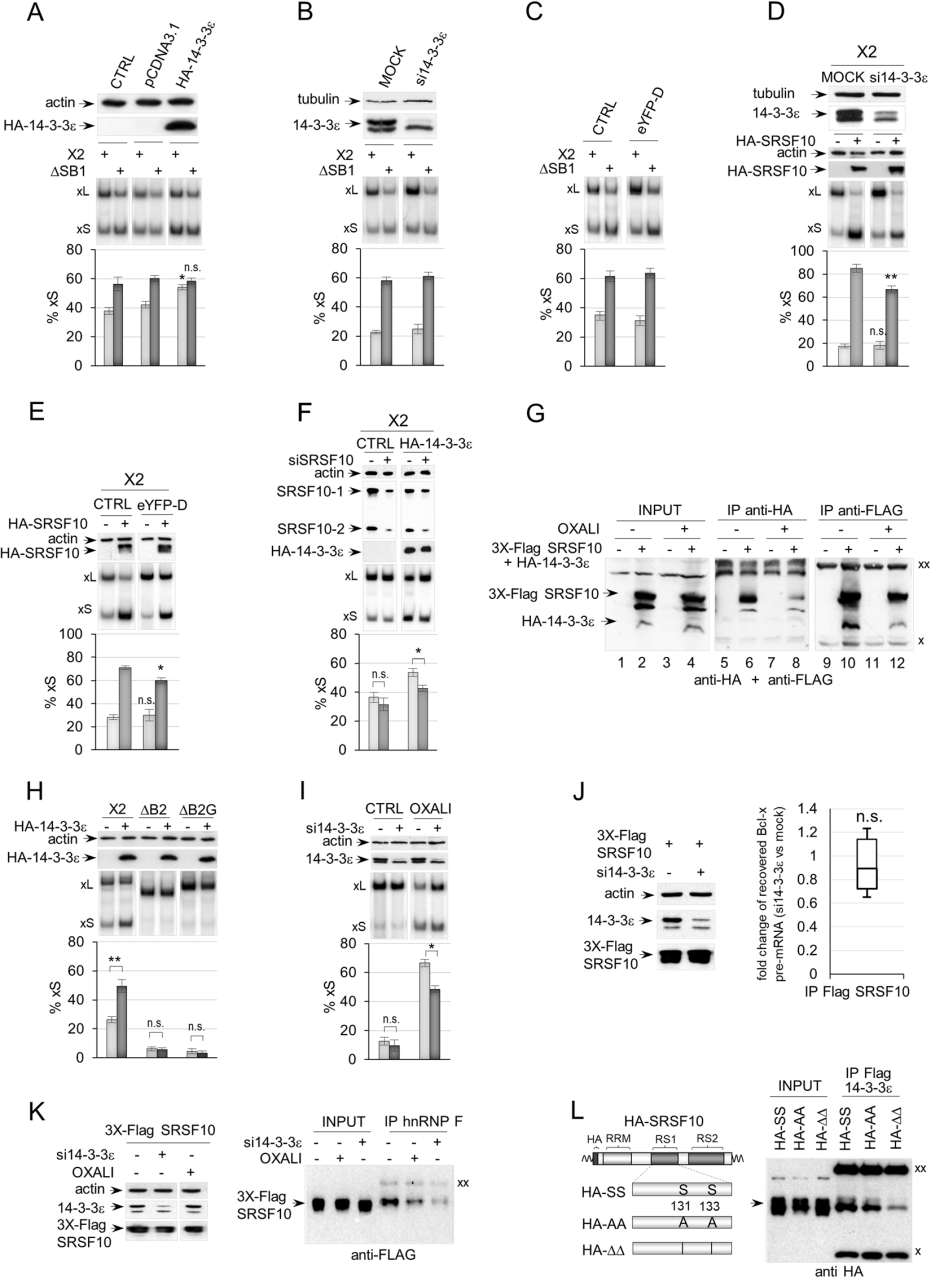
14-3-3ε modulates *Bcl-x* splicing and its response to DNA damage. (**A**) A plasmid programmed to express HA-14-3-3ε was co-transfected with minigene X2 or ∆SB1. Expression of HA-14-3-3ε was confirmed by immunoblots shown on *top*. Detection of actin was used as a loading control. The impact on *Bcl-x* splicing was monitored by RT-PCR assays using a minigene-specific pair of primers; radiolabeled RT-PCR products are shown for one experiment with the positions of the Bcl-xS and Bcl-xL products indicated. Histograms represent the average production of Bcl-xS in percentage from triplicates with standard deviations. The CTRL samples were transfected with the *Bcl-x* minigenes only, and pCDNA3.1 is an empty expression plasmid. (**B**) Cells were treated with si14-3-3ε, and an immunoblot was performed with anti-14-3-3ε antibodies (*top*). Detection of tubulin was used as a loading control. The *middle* panel shows one representative RT-PCR analysis of *Bcl-x* splicing, while the *bottom* panel displays the production of Bcl-xS from triplicate experiments. (**C**) Same as in (**B**) except that difopein (eYFP-D) was used to inactivate 14-3-3 proteins. (**D**) Cells treated or not with si14-3-3ε were transfected with HA-SRSF10 and minigene X2. Immunoblots on *top* display the depletion of 14-3-3ε and the expression of HA-SRSF10. (**E**) Same as in (**D**) except that eYFP-D was used to inactivate 14-3-3 proteins. *F*, SRSF10 contributes to the activity of 14-3-3ε. Minigene X2 was used to follow the impact of depleting SRSF10 with siSRSF10 when HA-14-3-3ε is ectopically expressed. (**G**) Immunoprecipitation assays using 293 cells treated or not with oxaliplatin and transfected or not with HA-14-3-3ε and FLAG-SRSF10. The material recovered was fractionated on gel and transferred on nitrocellulose decorated with anti-FLAG and anti-HA antibodies. (**H**) RT-PCR assays on *Bcl-x* after cells were transfected with *Bcl-x* minigenes, X2, ∆B2 and ∆B2G (the position of the B2 and B2G elements is shown in Fig. 1A) along with the HA-14-3-3ε expression plasmid. Expression of HA-14-3-3ε was confirmed by immunoblotting (shown on *top*). (**I**) Cells treated with the siRNA against 14-3-3ε were incubated or not with oxaliplatin and a RT-PCR assay was carried out to determine the impact of these treatments on the production of endogenous Bcl-xS. Immunoblots confirming the depletion are shown on *top*. (**J**) Impact of depleting 14-3-3ε on SRSF10 binding to the *Bcl-x* transcript. Immunoprecipitations with anti-Flag antibody were carried out on cells treated or not with si14-3-3ε and transfected with Flag-SRSF10. RNA was quantitated for *Bcl-x* pre-mRNA using primers shown in Fig. 3F and procedures described in the legend of Fig. 3F. (**K**) Impact of depleting 14-3-3ε on the interaction between hnRNP F and SRSF10. Immunoprecipitations using cells transfected with Flag-SRSF10 and with or without si14-3-3ε were carried out with anti-hnRNP F antibody and recovery of Flag-SRSF10 was monitored by immunoblotting. Oxaliplatin treatment was used as a control. (**L**) Interaction of mutated SRSF10 with 14-3-3ε. Cells transfected with Flag-14-3-3ε and wild-type (HA-SS), alanine-substituted (HA-AA) or serine-deleted (HA-∆∆) HA-SRSF10 at positions 131 and 133 were used in immunoprecipitation assays with the anti-Flag antibody. Recovered proteins were detected by immunoblot with the anti-HA antibody. In all cases, error bars indicate SD, and asterisks represent significant *P* values (two-tailed Student’s *t* test) comparing the means between samples and their respective controls. *P < 0.05, **P < 0.01 and ***P < 0.001; n.s. = not significant. In immunoblots, “xx” and “x” respectively indicate the large and small immunoglobulin subunits that react with the secondary antibody. Gels and blots cropped from different parts of the same gels/blots are indicated by white space between sections in panels A-I and K.

Because recovery of 14-3-3ε was associated with the presence of a splicing silencer element, we anticipated 14-3-3ε to repress the production of Bcl-xS. In contrast, overexpression of HA-tagged 14-3-3ε stimulated the production of Bcl-xS, and this stimulation was dependent on the presence of the SB1 element (Fig. 2A). The siRNA-mediated knockdown of 14-3-3ε, or the expression of a difopein peptide (eYFP-D), a specific inhibitor of 14-3-3/ligand interaction^36^, did not noticably affect the splicing of *Bcl-x* transcripts expressed from a minigene (Fig. 2B and C) or endogenously produced *Bcl-x* (see Fig. 2I). Given that the normal level of the Bcl-xS mRNA variant is relatively low, a drop may not be an easy event to promote and/or detect, especially since 14-3-3ε is a member of a large family of proteins that may functionally compensate for the loss of 14-3-3ε.

14-3-3ε is known to associate with SRSF10^31^, and SRSF10 stimulates the production of Bcl-xS^18^. Thus, 14-3-3ε may collaborate with SRSF10 to modulate *Bcl-x* splicing. This hypothesis was validated by showing that the siRNA-mediated depletion of 14-3-3ε compromised the HA-SRSF10-mediated increase in Bcl-xS (Fig. 2D). A similar result was obtained with difopein (Fig. 2E). A corrolary test of this collaborative interaction is to ask if the activity of 14-3-3ε if affected by depleting SRSF10. Using a level of depletion of SRSF10 that had no effect in normal conditions, the impact of ectopically expressed 14-3-3ε was nevertheless compromised by the depletion (Fig. 2F), consistent with the view that 14-3-3ε collaborates with HA-SRSF10 to stimulate the 5′ss of Bcl-xS.

To confirm the existence of an interaction between 14-3-3ε and SRSF10, we co-expressed HA-14-3-3ε and Flag-SRSF10 in 293 cells, and carried out an immunoprecipitation with an anti-HA antibody in cellular extracts treated with RNAse A. Immunoblot analysis of the recovered material revealed the presence of Flag-SRSF10 in the complexes (Fig. 2G, lane 6). The converse immunoprecipitation with the anti-Flag antibody recovered HA-14-3-3ε (Fig. 2G, lane 10). SRSF10 acts through an element (B2G) bound by the hnRNP F/H proteins, which interact with SRSF10 and are required for SRSF10 activity^18^. The B2G element was required to detect the stimulatory effect of HA-14-3-3ε (Fig. 2H), further validating that the activity of 14-3-3ε is linked to SRSF10.

We next asked if 14-3-3ε was important for SRSF10 to associate with the *Bcl-x* pre-mRNA in normal growth conditions. As shown in Fig. 2J, the depletion of 14-3-3ε did not affect the interaction of SRSF10 with the *Bcl-x* pre-mRNA, possibly indicating that 14-3-3ε instead modulates the interaction of SRSF10 with another splicing factor. We tested the effect of depleting 14-3-3ε on the known SRSF10/hnRNP F interaction that is disrupted when cells are treated with oxaliplatin^18^. We observed that the depletion of 14-3-3ε elicited a drop in the recovery of Flag-SRSF10 with the anti-hnRNP F antibody (Fig. 2K). 14-3-3ε protects SRSF10 from dephosphorylation^31^. Oxaliplatin promotes the dephosphorylation of SRSF10 at serine 133 and decreases the interaction between SRSF10 and hnRNP F (ref.^18^ and Fig. 2K). Deleting serine 133 and nearby serine 131 decreases the interaction between SRSF10 and hnRNP F^18^. These deletions (and to a lesser extent their mutations into alanines) also decreased the interaction of SRSF10 with 14-3-3ε (Fig. 2L). Overall, our results suggest that 14-3-3ε is required to foster the SRSF10/hnRNP F interaction that stimulates the 5′ss of Bcl-xS.

**Figure 3:**
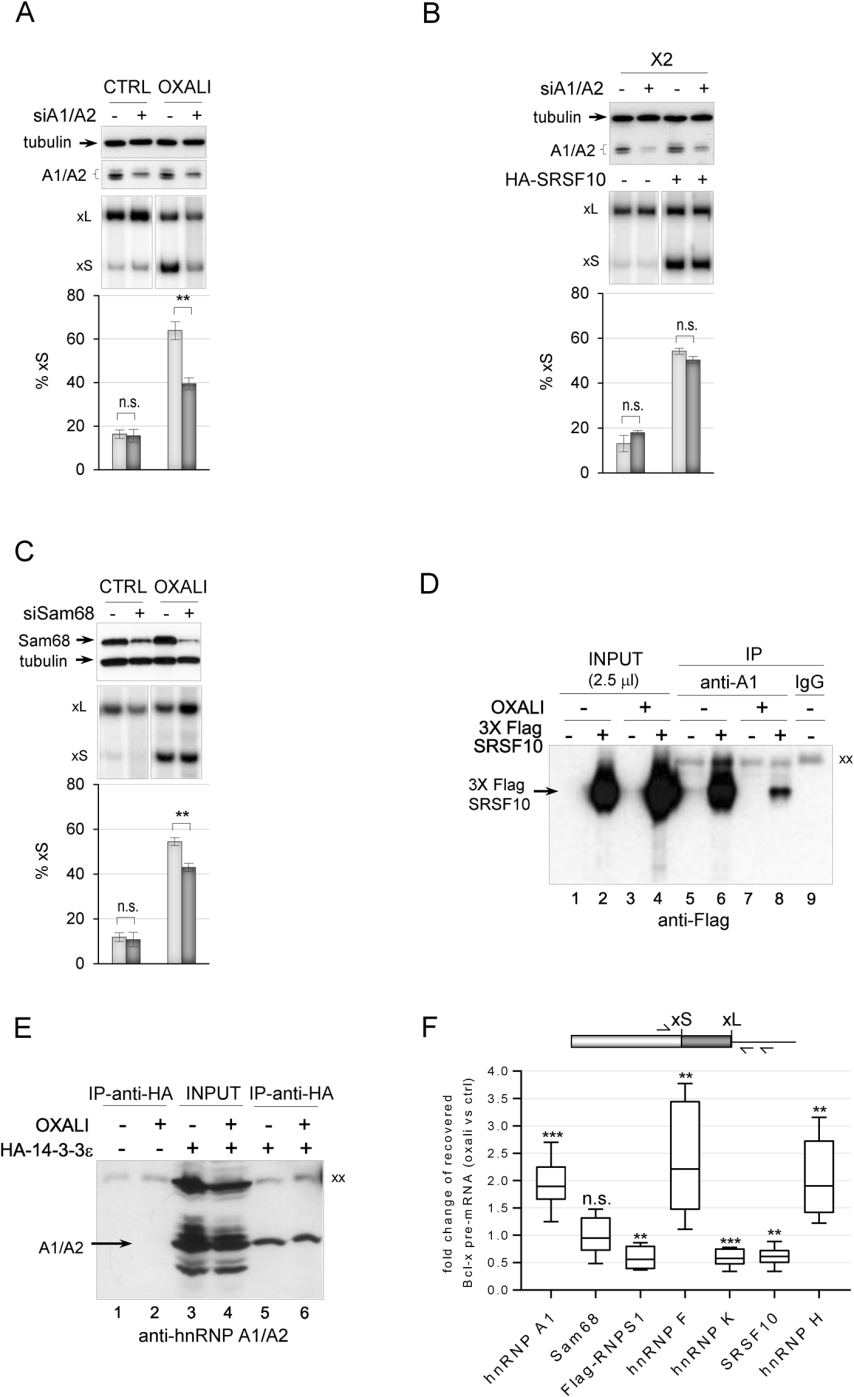
The depletion of hnRNP A1/A2 and Sam68 impairs the DDR modulation of *Bcl-x* splicing. (**A**) RT-PCR assays on endogenous *Bcl-x* transcripts using total RNA extracted from 293 cells transfected with siA1/A2 (96 hours). Cells were treated with or without 25 μM of oxaliplatin for the last 22 hours. The immunoblot analysis is shown, with tubulin as a loading control. (**B**) RT-PCR assays on X2 transcripts using total RNA extracted from 293 cells transfected with the X2 minigene, siA1/A2 and HA-SRSF10. The immunoblot analysis of the depletion is shown. (**C**) RT-PCR assays on endogenous *Bcl-x* transcripts using total RNA extracted from 293 cells transfected with siSam68 (72 hours) and treated with or without 25 μM of oxaliplatin for the 22 hours of that period. The immunoblot analysis to confirm the depletion of Sam68 is shown. In panels (**A**–**C**) the percentage of the Bcl-xS variant is plotted based on experiments performed in triplicates. (**D**) Anti-hnRNP A1 immunoprecipitation assays using 293 cells treated or not with oxaliplatin and transfected or not with FLAG-SRSF10. The material recovered was fractionated and transferred on nitrocellulose decorated with anti-FLAG antibodies. (**E**) Immunoprecipitation assays using 293 cells treated or not with oxaliplatin and transfected or not with HA-14-3-3ε. The material recovered was fractionated and transferred on nitrocellulose decorated with anti-hnRNP A1/A2 antibodies. “xx” and “x” respectively indicate the large and small immunoglobulin subunits that react with the secondary antibody. (**F**) Immunoprecipitations were carried out on cells treated or not with oxaliplatin. The recovered RNA was quantitated for *Bcl-x* pre-mRNA using primers shown on *top*. Raw data and control immunoprecipitations with IgG are provided in Supplementary Table S1. The box plots show the fold change of *Bcl-x* pre-mRNA recovered from the oxaliplatin-treated samples versus the non-treated control observed in three independent assays. Boxes indicate the median, the upper and lower quartiles, while whiskers display minimum and maximum values. The *P* values were determined by performing one-sample *t* test (compared to the hypothetical value of 1 (no change)) using GraphPad Prism. In panels A–C, error bars indicate SD. Asterisks represent significant *P* values (two-tailed Student’s *t* test) comparing the means between samples and their respective controls. *P < 0.05, **P < 0.01 and ***P < 0.001; n.s. = not significant. Gels and blots cropped from different parts of the same gels/blots are indicated by white space between sections in panels A–C.

Oxaliplatin elicits a shift in splicing to the 5′ss of Bcl-xS that requires ATM/CHK2^35^. SRSF10 is required for this oxaliplatin-induced splicing shift^18^. Oxaliplatin promotes the dissociation of SRSF10 and hnRNP K from hnRNP F/H and the *Bcl-x* pre-mRNA^18^. Notably, the siRNA-mediated depletion of 14-3-3ε reduces the impact of oxaliplatin on the splicing of endogenous *Bcl-x* transcripts (Fig. 2I). Thus, whereas the depletion of 14-3-3ε has little effect in normal growth conditions, 14-3-3ε appears to play a more important role when the DNA damage response pathway is activated. SRSF10 becomes dephosphorylated when cells are treated with oxaliplatin, and this change is associated with a loss in the interaction of SRSF10 with hnRNP F/H proteins and the *Bcl-x* pre-mRNA^18^. Using cells that co-express Flag-SRSF10 and HA-14-3-3ε, we noted that oxaliplatin decreased the recovery of Flag-SRSF10 with the anti-HA antibody (Fig. 2G, compare lane 8 with lane 6). Likewise, the recovery of HA-14-3-3ε with the anti-Flag antibody indicated a slight decrease (Fig. 2G, compare lane 12 with lane 10). The oxaliplatin-associated drop in interaction between 14-3-3ε and SRSF10 is in line with the observation that another DNA damaging agent (e.g. UV) promotes the dissociation of 14-3-3ε from SRSF10^37^.

### hnRNP A1/A2 and Sam68 are required for the *Bcl-x* splicing response to DNA damage

Although the depletion of hnRNP A1 by itself does not impact *Bcl-x* splicing, A1 is required for the activation of the 5′ss of Bcl-xS when Sam68 is overexpressed^12^. The siRNA-mediated depletion of both hnRNP A1 and A2 from 293 cells grown in normal conditions also had no impact on endogenous and X2-derived *Bcl-x* splicing, and did not affect the stimulatory activity of HA-SRSF10 (Fig. 3A,B). Likewise, the siRNA-mediated depletion of Sam68 had little impact (Fig. 3C), similar to a previous study^12^. We tested whether the contribution of hnRNP A1/A2 and Sam68 would be more apparent when repression at the 5′ss of Bcl-xS is lifted by oxaliplatin. As shown in Fig. 3A and C, the siRNA-mediated knockdown of A1/A2 or Sam68 compromised the oxaliplatin-induced splicing shift of endogenous *Bcl-x*, indicating that hnRNP A1/A2 and Sam68 are co-opted to activate the 5′ss of Bcl-xS when DNA damage occurs. As oxaliplatin promotes the dissociation of SRSF10 from the *Bcl-x* pre-mRNA and from hnRNP F/H but not hnRNP K^18^, we inquired about the interaction between SRSF10 and hnRNP A1. Immunoprecipitation assays indicated that hnRNP A1 interacts with SRSF10, but that this interaction was strongly reduced when cells are treated with oxaliplatin (Fig. 3D, compare lane 8 with lane 6 and see Supplementary Fig. 3A for the equivalent recovery of hnRNP A1). Given that both 14-3-3ε and hnRNP A1/A2 were recovered in association with the SB1 element, we asked whether they interact with one another. Immunoprecipitations carried out with the anti-HA antibody in cells expressing HA-14-3-3ε recovered hnRNP A1/A2, and this interaction was maintained when cells were treated with oxaliplatin (Fig. 3E). Ectopic expression of Myc-hnRNP A1 did not affect *Bcl-x* splicing whereas GFP-Sam68 shifted splicing to favor Bcl-xS in a SB1-dependent manner but independently of 14-3-3ε (Supplementary Fig. S3B and C).

To address whether oxaliplatin affects the interaction of hnRNP A1 and Sam68 with the *Bcl-x* pre-mRNA, we used quantitative RT-PCR to measure the amount of *Bcl-x* RNA recovered by immunoprecipitation with antibodies against hnRNP A1 and Sam68. We used a reverse transcriptase primer and one PCR primer that mapped in the intron downstream of the Bcl-xL 5′ss to ensure that we monitored interactions with the pre-mRNA. The recovered material was treated with DNase I to eliminate the potential contribution of contaminating genomic DNA. As shown in Fig. 3F and Supplementary Table S1, treating cells with oxaliplatin increased the association of hnRNP A1 with the *Bcl-x* pre-mRNA, similar to what we observed previously with hnRNP F and hnRNP H^18^, and reproduced here. Oxaliplatin had no significant impact on the interaction of Sam68 with the *Bcl-x* pre-mRNA (Fig. 3F). As observed previously, the interaction of SRSF10 and hnRNP K with the Bcl-x pre-mRNA decreased upon treating cells with oxaliplatin^18^. Thus, although hnRNP A1 and Sam68 do not control *Bcl-x* splicing in normal growth conditions, they may be part of a complex that interact with the *Bcl-x* pre-mRNA. Given that hnRNP A1 was recovered as a SB1-interacting factor, and that the *Bcl-x* splicing response to DNA damage requires the SB1 element, we also inquired about RNPS1, a repressor of Bcl-xS that we previously identified as acting through SB1^27^. The interaction of RNPS1 with the *Bcl-x* pre-mRNA decreased following treatment with oxaliplatin (Fig. 3F).

**Figure 4:**
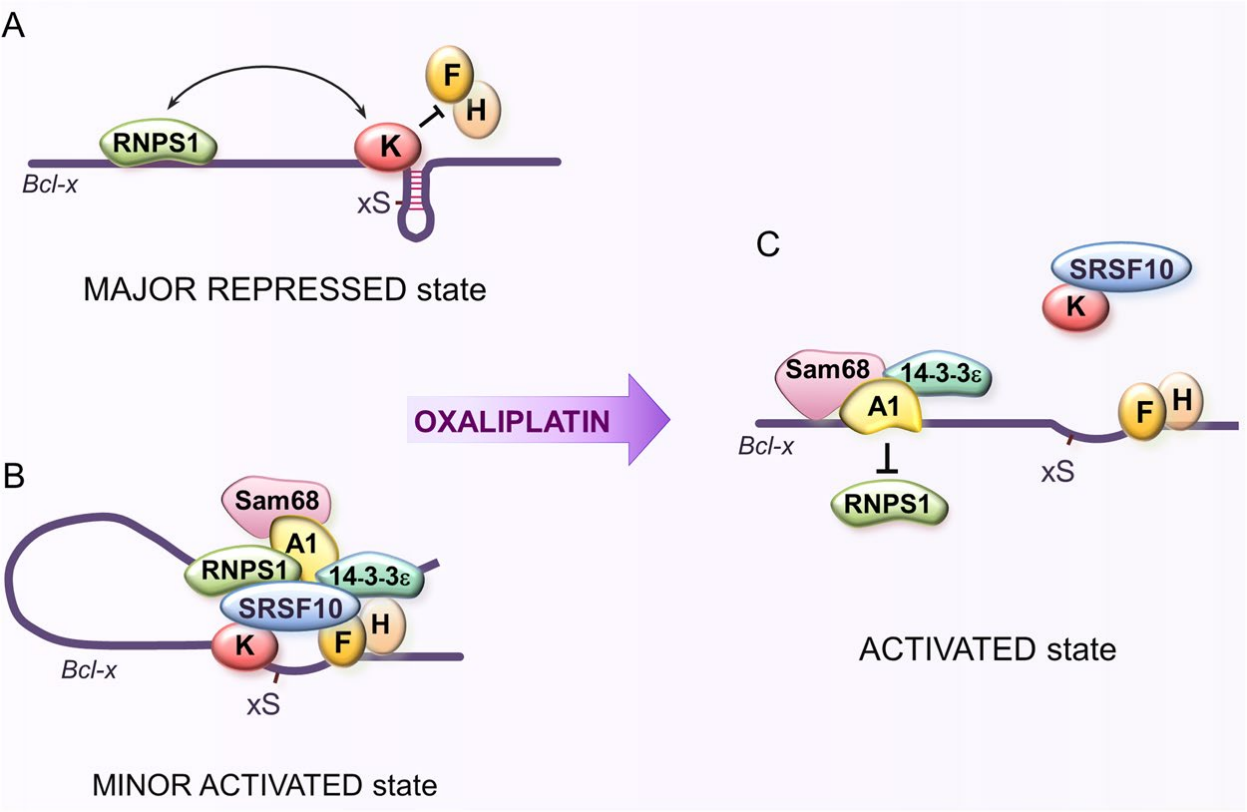
*Bcl-x* pre-mRNA splicing control is rewired by DNA damage. (**A**) Repressor complex representing the major regulatory assembly in normally growing 293 cells. (**B**) Activating complex proposed to represent a minor fraction of *Bcl-x* transcripts in normal growth conditions. (**C**) Activated complex assembled upon DNA damage. For simplicity, A1 is used to indicate hnRNP A1/A2 proteins (see text for details).

Based on the above results, we propose the following update to our model of *Bcl-x* splicing regulation (Fig. 4). Under normal growth conditions, hnRNP K represses the 5′ss of Bcl-xS on a majority of transcripts by antagonizing the binding of hnRNP F/H whose role is to render the 5′ss of Bcl-xS structurally available^20,21^ (Fig. 4A). The upstream SB1 region also contributes to this repression, and RNPS1 is implicated in this activity^27^, possibly through an interaction with hnRNP K. On a minor fraction of transcripts, a complex made up of SRSF10 and 14-3-3ε would antagonize the function of hnRNP K and RNPS1 by recruiting or stabilizing the binding of hnRNP F/H to favor 5′ss recognition (Fig. 4B). This model takes into account the positive function of 14-3-3ε, its recovery with the SB1 element and the interaction with SRSF10, itself interacting with hnRNP F/H at the B2G element. hnRNP A1/A2 and Sam68, may associate with this anti-repressor complex but their role at this stage may be negligible. Oxaliplatin, by triggering the dissociation of 14-3-3ε from SRSF10, would promote the dephosphorylation of SRSF10, a process that weakens the interaction of SRSF10 with hnRNP F/H^18^. Oxaliplatin decreases the association of both SRSF10 and hnRNP K with the *Bcl-x* transcript but not the interaction of hnRNP K with SRSF10^18^ (Fig. 4C). On the other hand, oxaliplatin promotes the displacement of RNPS1, possibly to be replaced by hnRNP A1 in association with Sam68 to prevent RNPS1 reassociation. Overall, the combined released of hnRNP K and RNPS1 would activate the 5′ splice site of Bcl-xS. It is unclear at this point what critical role 14-3-3ε accomplishes after DNA damage since it is required for the oxaliplatin-mediated splicing shift but not for Sam68 activity.

### hnRNP A1/A2 and Sam68 cooperate with SRSF10 to control a broader program of splicing response to DNA damage

**Figure 5:**
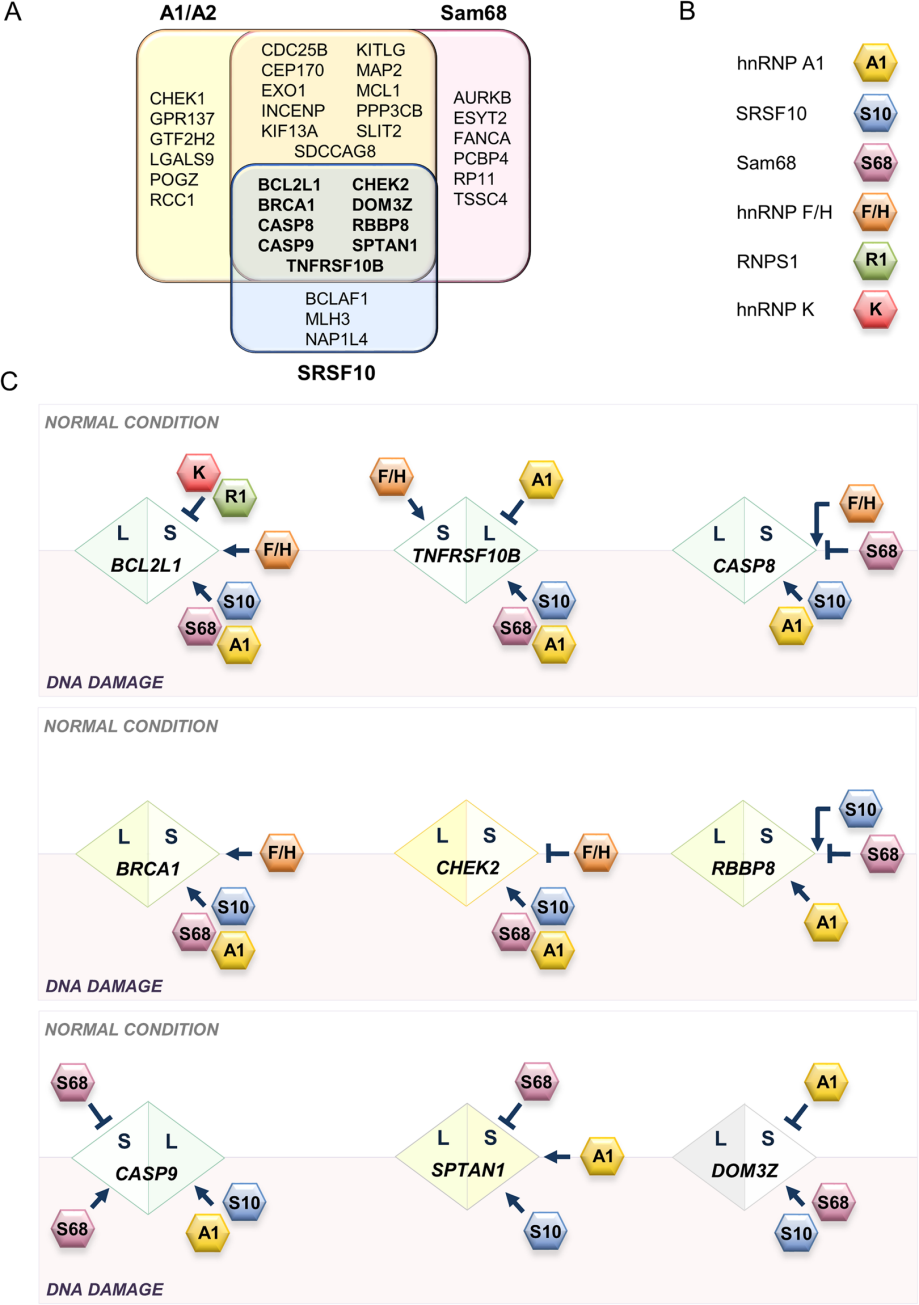
Control the splicing response to DNA damage by hnRNP A1/A2, Sam68 and SRSF10. (**A**) Summary of the oxaliplatin-sensitive alternative splicing events (ASE) that were reactive to the depletion of hnRNP A1/A2, Sam68 and SRSF10. ASEs reactive to all three are indicated in bold. (**B**,**C**) Diagrams representing splicing outcome and control strategies for the 9 ASEs that are controlled by SRSF10, hnRNP A1/A2 and Sam68 in normal and DNA damage conditions. The diamond contains the name of gene with the S and L half-portions respectively indicating the short and long variants. The direction of the splicing shift upon DNA damage is always from left to right, hence short to long for the CASP9 ASE but long to short for the DOM3Z ASE. RBPs implicated in splicing control are based on this study and reference^18^. RBPs in the above white area act during normal conditions. RBPs in the gray area are active upon DNA damage. RBPs overlapping both areas contribute to splicing control in both conditions. The impact of RBPs on the production of the L and S variants is indicated. For simplicity, A1 is used to indicate hnRNP A1/A2 proteins.

Given the role of hnRNP A1/A2 and Sam68 in enforcing the production of the Bcl-xS pro-apoptotic splice variant in response to DNA damage, we inquired about the contribution of these RBPs in the control of other transcripts whose alternative splicing are also affected by DNA damage in 293 cells. First, we identified 36 alternative splicing events (ASEs) from apoptosis, DNA repair and cell-cycle genes that reacted to oxaliplatin with a splicing shift greater than 10 percentage points (∆PSI > |10|, *P* value < 0.05; Supplementary Tables S2 and S3, OXALI-CTRL column). Of these oxaliplatin-sensitive ASEs, 26 were sensitive to the depletion of hnRNP A1/A2 (∆PSI > |5|, *P* value < 0.05; Supplementary Fig. S4 and Supplementary Table S2). We repeated the assay for Sam68, and identified 26 of 35 oxaliplatin-sensitive ASEs that were sensitive to its depletion (∆PSI > |5|, *P* value < 0.05; Supplementary Fig. S5 and Supplementary Table S3). Strikingly, ~75% of the oxaliplatin-sensitive ASEs regulated by hnRNP A1/A2 were also controlled by Sam68 (Fig. 5A). Of the 12 oxaliplatin-sensitive ASEs regulated by SRSF10 that we previously identified^18^, 9 were controlled by both hnRNP A1/A2 and Sam68 (Fig. 5A; Supplementary Fig. S6 and Supplementary Table S4). It is unclear how many of these targets are directly regulated by the above RBPs. Putative binding sites for Sam68, hnRNP A1 and SRSF10 are found in many co-regulated units, and validation of an interaction by eCLIP for Sam68 and hnRNP A1/A2 exist for several units, albeit in different human cell lines (Supplementary Table S5). Thus, we have uncovered a multicomponent regulatory hub made up of SRSF10, hnRNP A1/A2 and Sam68 that coordinates the splicing response to DNA damage of transcripts involved in apoptosis, cell-cycle control and DNA repair.

How DNA damage reconfigured splicing regulation very much depended on the identity of the transcripts and the regulatory RBP (Fig. 5C; Supplementary Figs S4–S6; Supplementary Tables S2–S4). While several depletion-responsive ASEs had a similar response both in oxaliplatin-treated and untreated cells (labeled as *same* in Supplementary Tables S2–S4), many ASEs, like *Bcl-x*, were more strongly affected when individual depletions were performed in oxaliplatin-treated cells (labeled as *co-opt* Supplementary Tables S2–S4). Other ASEs lost their reactivity to the depletion of some RBPs in oxaliplatin-treated cells (labeled as *anti* in Supplementary Tables S2–S4). Finally, a few ASEs shifted in the opposite direction when the impact of the depletion was compared between untreated versus oxaliplatin-treated cells (labeled as *reconf* in Supplementary Table S2–S4). In Fig. 5B,C, we are presenting a way of illustrating the contribution of multiple regulators in normal growth conditions and after DNA damage. Regulatory strategies between different ASEs can be easily compared both in terms of direction of splicing shift and regulatory RBPs. For several ASEs, depleting hnRNP A1/A2 and Sam68 suggests a collaborative action, while for others, the direction of the shift indicates an antagonistic relationship.

## Discussion

### Combinatorial control of *Bcl-x* splicing

SB1 is a regulatory element that represses the 5′ss of Bcl-xS located more than 150 nt downstream. Using different RNA-based affinity assays, we isolated 14-3-3ε and hnRNP A1/A2 in association with SB1. These proteins turned out to control positively the use of the Bcl-xS 5′ss, suggesting that they are part of an anti-repressor complex (Fig. 4C). The stimulating activity of 14-3-3ε is in line with the known interaction that 14-3-3ε entertains with SRSF10, also a stimulator of the 5′ss of Bcl-xS^18^. In normal growth conditions, SRSF10 interacts with hnRNP F/H on a minor subset of *Bcl-x* pre-mRNAs to offset repression imposed by hnRNP K, and RNPS1 bound to SB1^19,27^. 14-3-3ε, by protecting SRSF10 from dephosphorylation^31^, may help consolidate this complex. Upon treatment with oxaliplatin, the SRSF10/14-3-3ε interaction weakens, possibly leading to the dephosphorylation of SRSF10 and its dissociation from hnRNP F/H and the *Bcl-x* pre-mRNA (Fig. 4). However, both SRSF10 and 14-3-3ε are important for the oxaliplatin-mediated *Bcl-x* splicing shift; SRSF10 may be preventing hnRNP K from reassociating with the transcript, whereas the continued interaction of 14-3-3ε with hnRNP A1 may contribute to its productive collaboration with Sam68. Our results add to the already strong evidence documenting 14-3-3 proteins as preferential interactors with proteins involved in the DNA damage response^38^.

hnRNP A1 has been described as an activator of Bcl-xS because it collaborates with Sam68 to promote the use of the 5′ss of Bcl-xS^12^. Although hnRNP A1/A2 and Sam68 do not make substantial contribution to *Bcl-x* splicing in normal growth conditions, these proteins become critical for activating Bcl-xS when cells are treated with oxaliplatin. Moreover, the interaction of hnRNP A1 with the *Bcl-x* pre-mRNA increases following DNA damage. Oxaliplatin may promote tyrosine dephosphorylation on Sam68 by counteracting the activity of the Fyn kinase, which is normally activated through PKC signaling^39–41^. A dephosphorylated Sam68 may then interact more productively with hnRNP A1 to activate the 5′ss of Bcl-xS^12^. This model would explain why the *Bcl-x* splicing response to oxaliplatin requires activation of tyrosine phosphatases^35^. Finally, our model suggests that the interaction of the negative regulator RNPS1 with the SB1 region may be displaced by hnRNP A1/Sam68 (Fig. 4C).

Our model implies that the splicing response to DNA damage leads to a dynamic rewiring of the function of regulatory RBPs on the *Bcl-x* pre-mRNA. Following DNA damage the minor anti-repressor function of SRSF10 becomes more important possibly because its maintained interaction with a dissociated hnRNP K allows a more productive binding of hnRNP F/H. On the other hand, hnRNP A1/A2 and Sam68, which play minor roles in normal growth conditions, are co-opted following DNA damage to neutralize the repressor activity imposed by the SB1 element.

### A network of DNA damage responsive alternative splicing events controlled by collaborating RBPs

**Figure 6:**
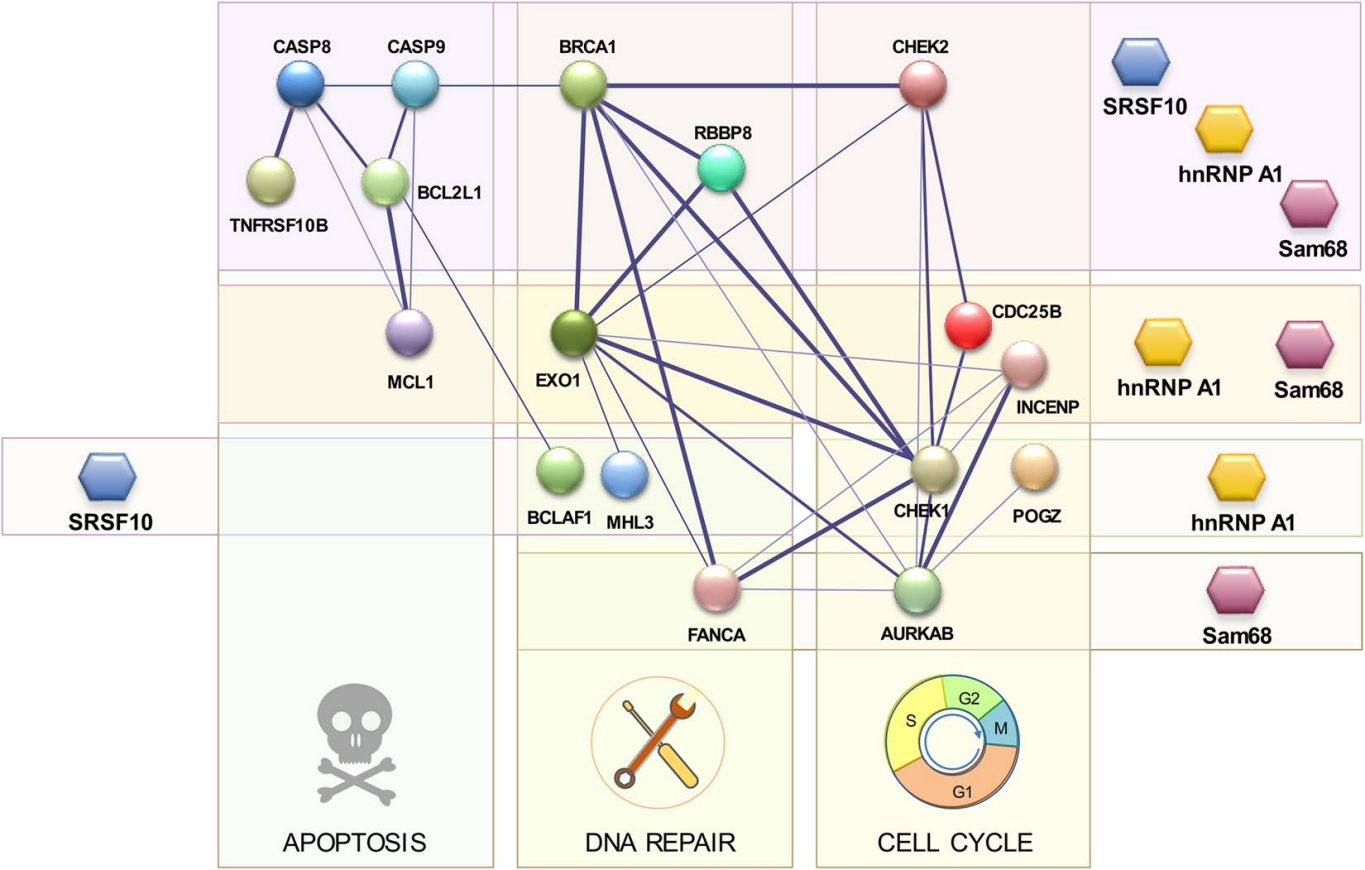
Splicing regulatory network. Graphical representation of splicing regulation of a functional interaction network of proteins involved in DDR. Circles (nodes) correspond to genes with ASEs that respond to oxaliplatin and that are regulated by hnRNP A1/A2, Sam68 and SRSF10. Lines (edges) indicate interactions and their thickness indicates confidence in data that support the interaction based on the STRING database^49^. Only components with connections are shown (18 of 34 of the oxaliplatin-sensitive ASEs regulated by the three RBPs). Target transcripts are organized vertically in groups that function in apoptosis, DNA repair and cell-cycle control; and horizontally, by individual or combinations of RBPs controlling their splicing.

Our study identified several RBPs involved in the *Bcl-x* splicing response to DNA damage. Because *Bcl-x* is an apoptotic regulator, it was of interest to determine whether this response was coordinated with other transcripts encoding factors involved in apoptosis, cell-cycle control and DNA repair. Our study reveals that approximately 25% (9 out of 37) of the alternative splicing events reacting to oxaliplatin are co-regulated by Sam68, hnRNP A1/A2 and SRSF10 (Fig. 5A). Our previous work showed that hnRNP F/H proteins further control at least four of these events (*BCL2L1, BRCA1, CHEK2* and *TNFRSF10B*)^18^. This core of regulators therefore displays extensive connectivity to the three DDR-critical pathways that are apoptosis, cell-cycle control and DNA repair (Fig. 6). In addition to this all-inclusive hub, coordinated regulation between hnRNP A1/A2 and Sam68 was operative for 11 more co-regulated DNA damage-responding events (Fig. 5A). Sam68, hnRNP A1/A2 and SRSF10 also regulate events that often involved products entertaining functional interactions. For example, MLH3 (regulated by SRSF10) interacts with EXO1 (regulated by Sam68 and hnRNP A1/A2); AURKB (regulated by Sam68) interacts with CDC25B and INCENP (regulated by Sam68 and hnRNP A1/A2). The picture that emerges from this analysis is that splicing factors act in concert to shape the production of a collection of splice variants involved in cell fate. While in a few cases, the contribution of the regulator remains similar following DNA damage, in most cases, a change in activity occurs. This reconfiguration either indicates co-opting, neutralization of function, or change of activity, from repressor to activator or vice-versa. For some splicing units, splicing regulatory factors collaborate to elicit a splicing change in the same direction (e.g. *BCL2L1, BRCA1, CHEK2, TNFRSF10B*), but in other units they antagonize one another (e.g. *CASP9, RBBP8*).

Many of the alternative splicing events that are responsive to DNA damage and that are regulated by hnRNP A1/A2 and/or Sam68 are likely to be functionally significant. The alternative splicing of *CHEK2, CASP8, CASP9, TNFRSF10B, DOM3Z, MCL1, FANCA, AURKB, CHEK1, GTF2H2* all involve exons that alter the reading frame (Supplementary Table S6). Other events are producing variants with known differences in function. For example, the tyrosine phosphatase CDC25B is important for G2/M checkpoints and mitotic progression, and the variant whose production is encouraged by oxaliplatin displays dominant negative activity^42^. In *KITLG*, Sam68 and hnRNP A1/A2 promote skipping of an exon that encodes a membrane-proximal extracellular protease cleavage site to yield the soluble form of KITLG rather than the membrane-associated variant^43,44^. In the DNA repair category, the DNA damage sensing protein *BRCA1* variant, whose production is stimulated by DNA damage and regulated by SRSF10, Sam68 and hnRNP A1/A2, lacks a linker region that separates the RING domain from a protein interaction platform. The hnRNP A1/A2-regulated alternative exon of GPR137 is also predicted to encode a transmembrane helix according to Exon Ontology^45^. As indicated in Fig. 6, several splicing events responsive to DNA damage occur in components involved in intra- and cross-functional category interactions. For example, the DNA repair factor RBBP8 codes for an endonuclease that controls cell-cycle checkpoints and interacts with BRCA1 to regulate the activation of the CHK1 kinase. CHK2 interacts with CHK1, CDC25B and the DNA repair factors EXO1 and BRCA1. These interactions suggest that the coordinated splicing response to DNA damage may amplify the functional outcome across different categories.

Overall, our study documents combinatorial strategies that control the production of splice variants involved in DNA repair, cell-cycle control and apoptosis. Coordinated regulation may occur through larger assemblies of regulatory proteins (LASR), as recently described for RBFOX proteins forming multimeric complexes with hnRNP proteins^46^. Our results suggest that the composition of such large complexes may be dynamic and may change upon DNA damage. The mechanism that rewires these activities during the DDR likely involves signaling. Oxaliplatin leads to the dephosphorylation of SRSF10 at residues that affect its activity and contribute to its interaction with other regulators^18^. Likewise, the oxaliplatin-mediated shift in *Bcl-x* splicing requires activation of tyrosine phosphatases whose target remain unclear^35^, but may include Sam68, a protein that loses it ability to regulate *Bcl-x* splicing when phosphorylated by the tyrosine kinase FYN1^12^. Future work will be important to determine how different stress pathways remodel distinct splicing regulatory complexes to modulate the production of functionally related group of effectors.

## Methods

### Construction of *Bcl-x* mutants

X2 and ΔSB1 minigenes (inserted into SVEDA-HIV-2 vector) were constructed as described previously^35^. The SB1 exhaustive mutagenesis transversion (changing A, G, C and T into T, C, G and A, respectively), deletion and linker (substitution of 10 nucleotides for GACTCAGTGT) mutants were produced by PCR site-directed mutagenesis, using the X2 minigene plasmid as template, the Pfu-turbo polymerase, and primers Human4 (ATGCCTGATCTCTGAAGCACAG) and PrHforw (TATAAATATACCCGCTCCGTGCA), with their associated mutation inserting primers. The derived PCR products were cleaved XhoI and KpnI and ligated into X2 minigene previously cut with the same enzymes. ∆SB1 + Reg9-19 minigene was produced by PCR amplification using the X2 minigene plasmid as PCR template, the Pfu-turbo polymerase, and primers AscI-8-rev (AGCTCTGGCGCGCCGCCTCAGTCCTGTTC) and AscI-25 (AGCTCTGGCGCGCCATGTCTCAGAGCAAC). The derived PCR product was cleaved AscI and ligated into ∆SB1 minigene previously cut with the same enzyme.

### MS2-based chromatography assay

The SB1 fragment or SB1∆3 derivative was tagged with ten repeats of the MS2 stem loop motif by PCR using the X2, X2-SB1∆3 minigenes and pTLC1-MS2x10 plasmid (generous gift of R. Wellinger’s laboratory) as templates and the following primers SB1-NheI-fwd (TTGCTAGCTAGGTCAGTCTCGAGCTT), SB1-MS2-rev (AATAATTTTTGGCATCCAAACTGCTGCTGTG), SB1-MS2-fwd (GCAGTTTGGATGCCAAAAATTATTCTAAATG) and MS2-EcoRI-rev (GCGAATTCTCCTAATGCCTTCGAT). The resulting SB1-10xMS2 and the SB1∆3-10xMS2 constructs were cleaved with NheI and EcoRI and ligated in pcDNA3.1. The MS2 coat protein fused to the TAP tag was made by PCR using plasmid pRS426-TAP-MS2pX2 and primers TAP-MS2-BamH-fwd (TAGGATCCATGGCAGGCCTTGCG) and TAP-MS2-XhoI-rev (CCCTCGAGGGCGTCATAGTAGAT) and was inserted into retroviral vector pMSCV.

pcDNA3-SB1-10xMS2 or pcDNA3-SB1∆3-10xMS2 and pMSCV-TAP-MS2pX2 were co-transfected in 293 cells that were treated with neomycin and hygromycin to select for stable clones expressing both MS2 tagged RNA and MS2 coat protein. Total cellular extracts prepared from selected clones were used for tandem affinity purification protocol, as described^47^. The final eluates were concentrated by TCA precipitation and were fractionated by SDS-PAGE. After silver staining, bands were cut out and analyzed by liquid chromatography-mass spectrometry (LC-MS/MS) (SickKids Hospital, Toronto).

### RNA-affinity chromatography

To prepare our RNA-affinity chromatography RNA targets, we first performed a cold transcription using T7 RNA polymerase. RNA substrates were synthesized *in vitro* from PCR products made of the X2 minigene Reg8-20 and the multiple cloning sites (MCS) region of pCDNA3.1+, using primers T7 B3 (TAATACGACTCACTATAGGTATTATAAAAATGT-CTC) and Region 20-18 reverse (CCCTTCTGGGTGTTCTCTTCCAC) for X2 and T7 (TAATACGACTCACTATAGGG) and RT4 (CTGATCAGCGGGTTTAAACG) for pCDNA3.1+. The *in vitro* transcription was performed at 37 °C for 4 hours in the following conditions: 1 μg DNA template, 4 mM rNTPs, 1×Frank’s buffer (10×: 400 mM Tris-HCl pH 7.9, 0.1% Triton X-100, 200 mM MgCl_2_, 20 mM spermidine), 10 mM DTT, 180 units of T7 RNA polymerase, 0.5 unit of yeast pyrophosphatase. At mid-point, an extra 180 units of T7 RNA polymerase was added to the reaction mix. Following migration in a 6% denaturating polyacrylamide gel (8 M urea), the transcripts were gel-extracted with Crunch Solution (0.2% SDS, 0.3 M sodium acetate), then phenol-chloroform-purified and ethanol precipitated.

As for the oxidation of the RNA targets, 1.5 nmol of gel-purified transcripts were incubated for 1 hour at room temperature, protected from light, in a 200 μL reaction volume containing 100 mM sodium acetate (pH 5.0) and 5 mM sodium meta-periodate (Sigma). The oxidized RNAs were ethanol-precipitated and resuspended in a 500 μL 100 mM sodium acetate (pH 5.0) solution. Adipic acid dihydrazide beads (Sigma) were washed and equilibrated in 100 mM sodium acetate (pH 5.0), making a 50% slurry solution. 200 μL of the equilibrated bead slurry was added to a 500 μL solution of oxidized transcripts and rotated overnight at 4 °C. Following washes with a 2 M NaCl solution, then with buffer DG (20 mM HEPES pH 7.9, 20% glycerol, 80 mM monopotassium glutamate, 0.2 mM EDTA, 0.2 mM PMSF and 1 mM DTT) with 60 mM NaCl, RNA coated beads were resuspended in 100 μL buffer DG+ 60 mM NaCl (50% slurry solution). HeLa nuclear extracts (250 μL) were added to the 100 μL RNA coated beads solution (final reaction volume increased at 1 mL with buffer DG+ 60 mM NaCl) and rotated overnight at 4 °C. Following 5 washes with buffer DG+ 60 mM NaCl solution, the bound proteins were stepwise eluted with buffer DG containing increasing concentrations of NaCl (100 mM, 200 mM, 400 mM and 1 M). These eluates were precipitated by adding 1 volume of trichloroacetic acid (TCA), incubating for 1 hour on ice, and spinning for 10 min at 4 °C (10 000× g). The pellets were washed with ice cold 0.01 M HCl+ 90% acetone and resuspended in 80 μL 0.1 N NaOH. Ten μL of the various eluates, in Laemmli buffer (2×: 20% glycerol, 10% β-mercaptoethanol, 4.6% SDS, 0.125 M Tris and 0.1% bromophenol blue) were loaded in a 10% polyacrylamide gel and, following migration, stained with silver nitrate, and the bands of interest were cut out and analyzed by LC-MS/MS (SickKids Hospital, Toronto).

### Transfection and cell treatment

Human 293 cells (ECR293, Invitrogen) were grown at 37 °C (5% CO_2_) in Dulbecco’s modified Eagle’s medium (DMEM) supplemented with 10% fetal bovine serum (FBS) and 1% glutamine (Wisent). Oxaliplatin was obtained from the Service Pharmaceutique du Centre de Chimiothérapie at the Centre Hospitalier de l’Université de Sherbrooke.

For plasmids transfections, 293 cells (4 × 10^5^) were plated in 35-mm^2^ wells. Twenty-four hours later, 1 μg of DNA and 5 μl of polyethyleneimine (1 μg/μl) were incubated for 20 min in 100 µl (final volume) of Opti-MEM before being added to the wells, which contained 1 mL of DMEM. Five hours later, 1 mL of DMEM with or without oxaliplatin was added to each well. Cells were harvested 18 h later, and RNA was extracted using TRIzol (Invitrogen).

For knockdowns, 293 cells were transfected using Lipofectamine 2000 (Invitrogen) with 80 nM of and anti-14-3-3ε siRNA (sc-29588, Santa Cruz Biotechnology. Inc. Mississauga, Ontario, Canada) an A1-specific siRNA (AAUGGGGAACGCUCACGGACUdTdT), an A2-specific siRNA (AACCACAGAAGAAAGUUUGAGdTdT) or a Sam68-specific siRNA (AUAACGUCCAUAUGGGUGCdTdT) purchased from IDT. 293 cells were treated for 96 hours with siA1/A2 and 72 hours with siSam68. Twenty-two hours pre-harvest, the cells were treated with or without 25 μM of oxaliplatin. Cells were also transfected with plasmid for enhanced YFP-fused-difopein expression (pSCM138; kindly provided by Dr. Haian Fu, School of Medicine, Emory University). Proteins and RNA were extracted to verify depletion through immunoblot analysis and endogenous *Bcl-x* splicing profiles using RT-PCR.

### RT-PCR assays

Total RNA was extracted from treated or transfected cells with TRIzol (Invitrogen) using the procedure described by the manufacturer. The splicing profile of *Bcl-x* was assessed by RT-PCR. Reverse transcription was done using the OmniScript RT kit (Qiagen) with random hexamers for endogenously derived *Bcl-x* mRNAs, whereas oligonucleotides RT-Sveda-Rev (GGGAAGCTAGAGTAAGTAG) was used for the SVEDA-2 plasmid-derived mRNAs and RT3 (GAAGGCACAGTCGAGGCTG) was used for the pcDNA 3.1+ plasmid-derived mRNAs. One fifth of cDNA material was used as template for the PCR. Primers X3 (ATGGCAGCAGTAAAGCAAGCG) and X2 (TCATTTCCGACTGAAGAGTGA) were used to amplify fragments of splicing isoforms derived from endogenous *Bcl-x*, whereas primers X34 (AGGGAGGCAGGCGACGGCGACGAGTTT) and X-Age-Rev (CTTACCGGTGGATCCCCCGGGCTGCAGGAATTCGAT) were used for the SVEDA-2 plasmid-derived cDNA and T7 (TAATACGACTCACTATAGGG) and RT4 (CTGATCAGCGGGTTTAAACG) were used for pCDNA3.1+ plasmid-derived cDNA. For the conventional PCR, [α-^32^P]dCTP (PerkinElmer Canada Inc.) was added to PCR mixtures, and amplification products were fractionated onto a 4% native polyacrylamide gel. Gels were exposed on screens that were scanned on a STORM PhosphorImager 860 (GE Healthcare). The intensity of the bands was quantified using the Image-Quant software.

As for the apoptotic and cell cycle control and DNA repair splicing units, RT-PCR were performed by the RNomics Platform of the Université de Sherbrooke. Primers are listed in Supplementary Table S6. Reverse transcription was done using 10 U of Transcriptor reverse transcriptase, 20 U of RNaseOUT (Invitrogen), 3.2 µg of random hexamers, 1 µM of dNTPs mix, 1× Transcriptor RT reaction buffer and 0.2–2 µg of total RNA. PCR was done using 0.2 U of Platinium Taq, 0.6 µM of primers, 1.5 mM of MgCl_2_, 10 ng of cDNA template, 1× of PCR buffer and 200 µM of dNTPs mix. PCR reactions were performed on thermocyclers GeneAmp PCR System 9700 (Thermo Scientific-Invitrogen). A first cycle of 15 min at 95 °C was followed by 35 cycles of 30 s at 94 °C, 30 s at 55 °C, and 1 min at 72 °C. The reaction was ended with the extension step of 10 min at 72 °C. Visualization and analysis of amplified products were done using automated chip-based microcapillary electrophoresis on Labchip GX Touch HT instruments (Perkin Elmer).

### Immunoblots and protein immunoprecipitation assays

To assess the efficiency of depletion, total proteins were extracted from mock treated and siRNAs treated cells and standard Western analysis was performed using anti-14-3-3ε (Biolegend, #637901), anti-hnRNP A1/A2 (custom made, CHUL, Ste-Foy, Québec)^48^ and anti-Sam68 (07–415, Upstate) antibodies. Recombinant proteins were revealed with primary antibodies against the HA-tag (Roche 12CA5), the FLAG-tag (Sigma F3165), actin (Sigma 2066), tubulin (ab4074, Abcam), using peroxidase-conjugated secondary antibodies and ECL detection reagent (Amersham). Secondary antibodies were either polyclonal anti-rabbit (Cell Signalling 7074) or anti-mouse (BioCan 115-035-003).

### RNA immunoprecipitation and RT-qPCR analysis

ECR293 cells treated with 20 µM of oxaliplatin for 24 hours. For monitoring RNPS1 and SRSF10, cells were transfected with Flag-RNPS1 and FLAG-SRSF10 24 hours before oxaliplaitn treatment. After washing with PBS, the cell pellet was resuspended into NET-2 buffer supplemented with protease and RNase inhibitors. Cells were lysed by sonication and the insoluble material was removed by centrifugation at 4 °C. The supernatant was precleared by incubation for 1 hour at 4 °C with SureBeads Protein G Magnetic beads (BioRad #161-4023) previously blocked with yeast tRNA. An aliquot of the precleared supernatant was used as input while the remaining material was used for immunoprecipitation. Precleared whole-cell lysates of equal protein quantities were incubated overnight at 4 °C with SureBeads Protein G Magnetic beads coated with antibodies against hnRNP F, H, K, A1 and Sam68 or against the Flag epitoipe for Flag-RNPS1 and Flag-SRSF10. Beads were washed 4 times with NET-2. RNA was extracted by applying TRIzol directly on washed magnetic beads. Extracted RNA was resuspended in 15 μL of H_2_O, treated with DNase I for 15 min at 37 °C, and quantitated by spectrometry. Equal quantities of RNA were reverse transcribed using Omniscript Reverse Transcriptase (Qiagen) enzyme and the primer X-Int2-1-REV (CAGAGGCCAAAGAAAAGGGACACA) annealing in intron 2 of *Bcl-x*. Quantitative PCR was carried out using SYBR green (2× Power SYBR green master mix; ABI 4367660) and primers X-Int2-2-REV (CACACAAGGGGCTTGGTTCTTA) and X-EX-S1-FWD (TCACCCCAGGGACAGCATATC). The method used to determine the relative abundance of *Bcl-x* pre-mRNA in immunoprecipitates compared Ct using the input sample (pre-immunoprecipitated) as reference, while the difference between control and oxaliplatin-treated samples was calculated using the 2^−ΔΔ*C*t^ method and was expressed as fold change of *Bcl-x* pre-mRNA recovered from oxaliplatin-treated samples versus the non-treated control.

## Electronic supplementary material

Supplementary Information
