# Supplementary material for: hnRNP A1/A2 and Sam68 collaborate with SRSF10 to control the alternative splicing response to oxaliplatin-mediated DNA damage
Source: Sci Rep. 2018 Feb 2;8:2206. doi: 10.1038/s41598-018-20360-x (PMC5797138; doi:10.1038/s41598-018-20360-x)
Supplement: Supplementary file 1 — Supplementary Information [file 41598_2018_20360_MOESM1_ESM.pdf]

SUPPLEMENTARY INFORMATION

hnRNP A1/A2 and Sam68 collaborate with SRSF10 to control the alternative splicing response to oxaliplatin-mediated DNA damage

Alexandre Cloutier, Lulzim Shkreta, Johanne Toutant, Mathieu Durand, Philippe Thibault and Benoit Chabot

SUPPLEMENTARY TABLES

Supplementary Table S1. RT-qPCR values for immunoprecipitation of *Bcl-x* pre-mRNA. The anti-hnRNP A1, anti-hnRNP F, anti-hnRNP H, anti-hnRNP K, anti-Sam68 and anti-Flag (to recover Flag-SRSF10 anf Flag-RNPS1) antibodies were used in extracts prepared from cells treated or not with oxaliplatin. RT-qPCR was performed on the same amount of RNA for input and immunoprecipitated material from both conditions (oxaliplatin-treated and untreated). Ct values for all tests are indicated. After calculating the ΔCt for each pair of IP/input, ΔΔCt values were calculated for oxaliplatin-treated/untreated pair, and their averages were used to obtain a fold-difference value, as described in Methods. A control antibody (IgG) was also used.

Supplementary Table S2. Oxaliplatin-sensitive ASEs that are regulated by hnRNP A1/A2. ASEs are ranked by order of sensitivity to oxaliplatin based on the average ΔPSI relative to the untreated controls. ASEs displaying a ΔPSI > |10| with a P value smaller than 0.05 are indicated in salmon color. The reactivity to the depletion of hnRNP A1/A2 are then indicated (in normal growth conditions or in oxaliplatin treated cells). ΔPSI >|5| with a P value < 0.05 are considered regulated by hnRNP A1/A2 and are compared when cells are treated or not with oxaliplatin. Differences in shifting behavior following the depletion of A1/A2 are indicated in column W: *same* indicates that ΔA1/A2 promotes a significant shift in the same polarity in both untreated and oxaliplatin-treated cells , *co-opt* indicates that the ΔA1/A2-mediated shift is seen only in oxaliplatin-treated cells, *anti* indicates that the ΔA1/A2-mediated shift seen in untreated cells is not occurring in oxaliplatin-treated cells, and *reconf* indicates that the polarity of the shift in untreated and oxaliplatin treated cells is in opposite direction. *SLIT2* was listed as a co-opted event because its sensitivity to the depletion of hnRNP A1/A2 was much greater in the presence

of oxaliplatin (ΔPSI of 17 percentage points in normal conditions, and 53 in cells treated with oxaliplatin). Standard deviations and *P* values for the differences are given. All assays were performed in triplicates.

Supplementary Table S3. Oxaliplatin-sensitive ASEs that are regulated by Sam68. ASEs are ranked by order of sensitivity to oxaliplatin based on the average ΔPSI relative to the untreated controls. ASEs displaying a ΔPSI > |10| with a *P* value smaller than 0.05 are indicated in salmon color. The reactivity to the depletion of Sam68 are then indicated (in normal growth conditions or in oxaliplatin treated cells). ΔPSI >|5| with a *P* value < 0.05 are considered regulated by Sam68 and are compared between growth conditions (with or without oxaliplatin). Differences in shifting behavior are indicated in column W; *same* indicates that ΔSam68 promotes a significant shift in the same polarity in both untreated and oxaliplatin-treated cells , *co-opt* indicates that the ΔSam68-mediated shift is seen only in oxaliplatin-treated cells, *anti* indicates that the ΔSam68-mediated shift seen in untreated cells is not occurring in oxaliplatin-treated cells, and *reconf* indicates that the polarity of the shift in untreated and oxaliplatin treated cells is in opposite direction. *INCENP* is listed as *anti* because its sensitivity to the depletion of Sam68 was much greater in cells grown under normal conditions than with oxaliplatin (ΔPSI moving from 36 to 6 percentage points). Standard deviations and *P* values for the differences are given. All assays were performed in triplicates.

Supplementary Table S4. Oxaliplatin-sensitive ASEs regulated by SRSF10. Results are taken from Shkreta et al. (2016). ASEs are ranked by order of sensitivity to oxaliplatin based on the average ΔPSI relative to the untreated controls. ASEs displaying a ΔPSI > |10| with a *P* value smaller than 0.05 are indicated in salmon color. The reactivity to the depletion of SRSF10

is then indicated (in normal growth conditions or in oxaliplatin treated cells). ΔPSI >|5| with a *P* value < 0.05 are considered regulated by SRSF10 and are compared between growth conditions (with or without oxaliplatin). Differences in shifting behavior are indicated in column W: *same* indicates that ΔSRSF10 promotes a significant shift in the same polarity in both untreated and oxaliplatin-treated cells, *co-opt* indicates that the ΔSRSF10-mediated shift is seen only in oxaliplatin-treated cells, and *anti* indicates that the ΔSRSF10-mediated shift seen in untreated cells is not occurring in oxaliplatin-treated cells. Standard deviations and *P* values for the differences are given. All assays were performed in triplicates.

Supplementary Table S5. Putative binding sites for hnRNP A1, Sam68 and SRSF10 in the 9 co-regulated units. For each alternative splicing unit, the size and coordinates of the region analyzed that include alternative and flanking sequences are given. When alternative regions contained several alternative exons, these regions were analyzed separately (e.g. caspases and BRCA1). Two platforms were used to search for binding motifs and the ENCODE project was used for eCLIP data for hnRNP A1 and Sam68 (references are given at bottom). For eCLIP data, the number of signal peaks in the relevant region is given followed by a visual assessment of the intensity of individual peaks (when only one number is given, all peaks have a similar value; e.g. 5;6 means 5 peaks of intensity 6; and 3;1-3-1 means 3 peaks of respective intensity of 3, 1 and 3).

Supplementary Table S6. Primers used for endpoint RT-PCR of various cellular splicing units and size of amplicons.

SUPPLEMENTARY FIGURE LEGENDS

Supplementary Figure S1. The SB1 element and its subregions. *A,* Wild-type sequence of the 31 subregions that form the SB1 element. *B,* Sequence of the transversion mutants for each of the 31 subregions.

Supplementary Figure S2. RNA binding assays used to recover proteins associating with SB1. *A*, Procedure for the recovery of SB1-interacting proteins by expressing a SB1-MS2 hybrid RNA and a control SB1Δ3-MS2 version. The SB1Δ3-MS2 RNA harbors deletions of segments Reg11, Reg17 and Reg23. *B*, Impact of the individual deletions on *Bcl-x* splicing as measured by RT-PCR. *C*, qRT-PCR was used to monitor the abundance of SB1-MS2 and SB1Δ3-MS2 in two stably expressing clones. *D*, An immunoblot analysis was performed to detect the co-expressed TAP-MS2 protein in the stably expressing clones of panel *C*. *E*, Silver-stained gel of recovered proteins following tandem affinity purification. The band analyzed by mass spectrometry is indicated. *F,* Schematic representation of the *in vitro* RNA-affinity chromatography assay. Acid adipic dihydrazide agarose beads were coated with oxydized RNA to which we added HeLa nuclear extracts. Bound proteins were eluted with increasing concentrations of NaCl. *G,* SDS-Page gel with 200 mM NaCl eluates and mass spectrometry results. The various eluates from the RNA-affinity chromatography assay were loaded on a 10 % SDS-polyacrylamide and stained with silver nitrate. The bands of interest were cut out and analysed by mass spectrometry. *H,* Based on the optimal site for hnRNP A1 TAG$^{A}/_{G}$GT [51], putative binding sites for hnRNP A1 and A2 are highlighted (in gray) within the target region (central repressive subregion of SB1).

Supplementary Figure S3. Impact of ectopic expression of GFP-Sam68 and Myc-hnRNP A1 on *Bcl-x* splicing. A. Immunoblot showing that hnRNP A1 was recovered in all samples in

a nearly equivalent manner. The immunoblot shown in Figure 3D was reprobed with the anti-A1 antibody. Another segment of the same gel/membrane containing more input material was used to localize hnRNP A1 proteins. The blot shown was cropped from different parts of the same blot as indicated by white space between the two sections. **B-C.** Plasmids programmed to express GFP-Sam68 and myc-hnRNP A1 were co-transfected with *Bcl-x* minigene X2 (panel B) or ΔSB1 (panel C). Expression of proteins was confirmed by immunoblots shown on *top*. Actin was used as a loading control. The impact on *Bcl-x* splicing was monitored by RT-PCR using a minigene-specific pair of primers; radiolabeled RT-PCR products are shown for an experiment in triplicate with the positions of the Bcl-xS and Bcl-xL products. Histograms represent the average production of Bcl-xS in percentage from triplicates with standard deviations. The CTRL samples was transfected with the *Bcl-x* minigenes only. Transfected 293 cells were also pre-treated with si14-3-3ε, an immunoblot was performed with anti-14-3-3ε antibodies (*top*).

Supplementary Figure S4. hnRNP A1/A2 hits. RT-PCR analysis of oxaliplatin reactive ASEs upon depletion of hnRNP A1/A2 in untreated and oxaliplatin-treated ECR293 cells. *A*, ECR293 cells were transfected with sihnRNP A1/A2 (96 hours). Cells were treated 22 hours with or without 25 μM oxaliplatin. Immunoblot analysis was performed with anti-A1/A2 antibodies to confirm the depletion. The histograms display percent splicing index (PSI). The sizes of expected RT-PCR products and the alternative exon are indicated in Supplementary Table S5. *P* values for differences are indicated in Supplementary Table S2. *A*. hnRNP A1/A2 hits shared with Sam68 and SRSF10 based on Figure 5A. *B*. hnRNP A1/A2 hits shared with Sam68. *C*. Hits unique to hnRNP A1/A2.

Supplementary Figure S5. Sam68 hits. RT-PCR analysis of oxaliplatin reactive ASEs upon depletion of Sam68 in untreated and oxaliplatin-treated ECR293 cells. The histograms display

percent splicing index (PSI). The sizes of expected RT-PCR products and the alternative exon are indicated in Supplementary Table S5. *P* values for differences are indicated in Supplementary Table S3. *A*. Sam68 hits shared with hnRNP A1/A2 and SRSF10 based on Figure 5A. *B*. Sam68 hits shared with hnRNP A1/A2. *C*, Hits unique to Sam68.

Supplementary Figure S6. SRSF10 hits. RT-PCR analysis of oxaliplatin reactive ASEs upon depletion of SRSF10. Data is taken from Shkreta et al. (2016). The histograms display percent splicing index (PSI). The sizes of expected RT-PCR products and the alternative exons are indicated in Supplementary Table S5. *P* values for differences are indicated in Supplementary Table S4.

A

5′- CTTCAGAATCTTATCTTGGCTTTGGATCTTAGAAGAGA**ATCACTAACC**AGAGACGAGA**CTCAGTGAGT**

1 2 3

GAGCAGGTGT**TTTGGACAAT**GGACTGGTTG**AGCCCATCCC**TATTATAAAA**ATGTCTCAGA**GCAACCGG

4 5 6 7 8 9 10

GA**GCTGGTGGTT**GACTTTCTCT**CCTACAAGCT**TTCCCAGAAA**GGATACAGCT**GGAGTCAGTT**TAGTGA**

11 12 13 14 15 16 17

TGTGGAAGAGAACA**GGACTGAGGC**CCCAGAAGGG**ACTGAATCGG**AGATGGAGAC**CCCCAGTGCC**AT

18 19 20 21 22 23

CAATGGCA**ACCCATCCTG**GCACCTGGCA**GACAGCCCC**GCGGTGAATG**GAGCCACTGG**CCACAGCAGC

24 25 26 27 28 29 30

A**GTTTGGATGCC** -3′

31

B

5′- CTTCAGAATCTTATCTTGGCTTTGGATCTTAGAAGAGA**TAGTGATTGG**TCTCTGCTCT**GAGTCACTCA**CT

1 2 3

CGTCCACA**AAACCTGTTA**CCTGACCAAC**TCGGGTAGGG**ATAATATTTT**TACAGAGTCT**CGTTGGCCCT**C**

4 5 6 7 8 9 10

GACCACCAACTGAAAGAGA**GGATGTTCGA**AAGGGTCTTT**CCTATGTCGA**CCTCAGTCAA**ATCACTACA**

11 12 13 14 15 16 17

CCTTCTCTTGT**CCTGACTCCG**GGGTCTTCCC**TGACTTAGCC**TCTACCTCTG**GGGGTCACGG**TAGTTACC

18 19 20 21 22 23 24

GT**TGGGTAGGAC**CGTGGACCGT**CTGTCGGGG**CGCCACTTAC**CTCGGTGACC**GGTGTCGTCGT**CAAAC**

25 26 27 28 29 30

CTACGG -3′

31

SUPPLEMENTARY FIGURE S1

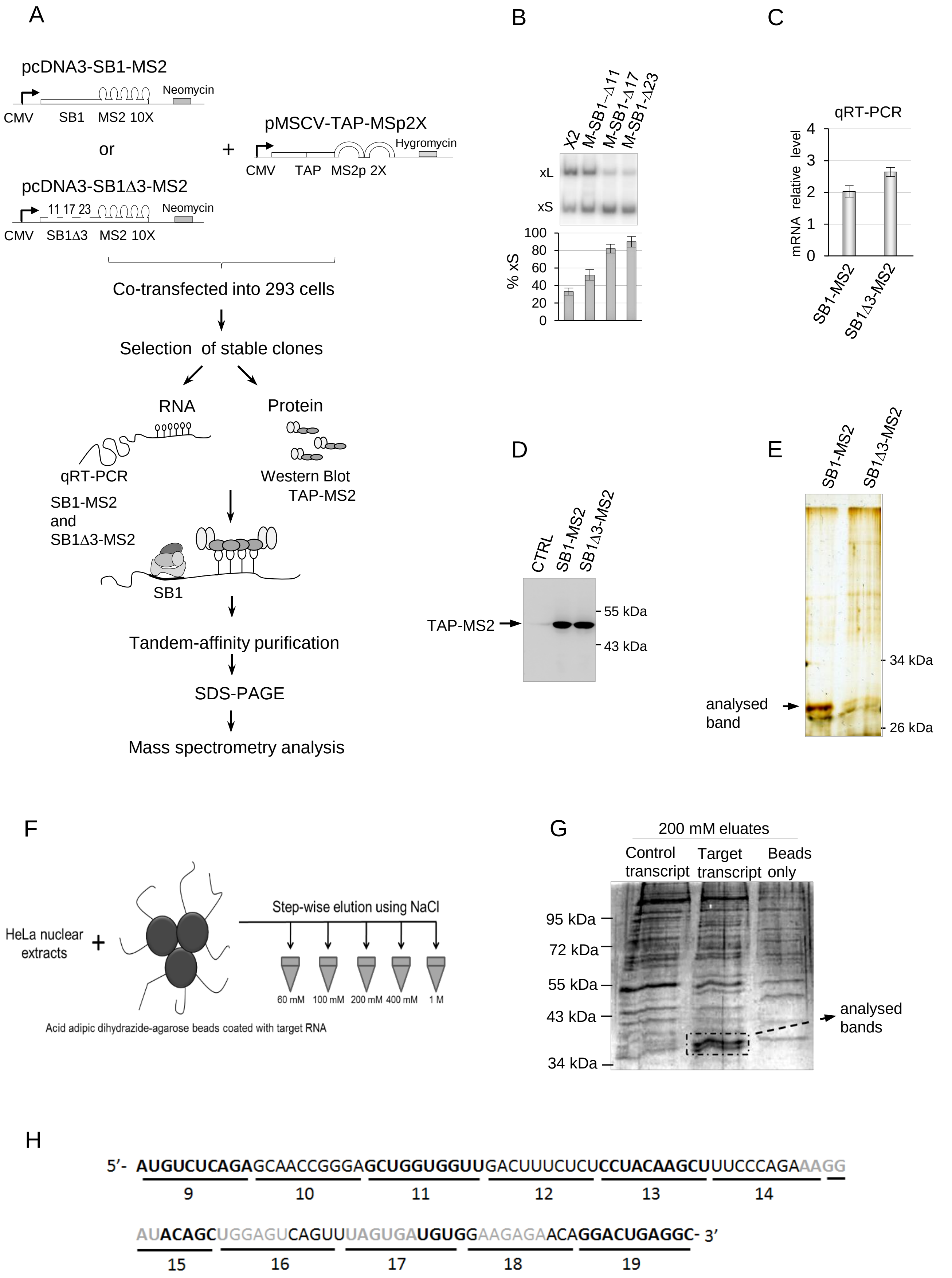

SUPPLEMENTARY FIGURE S2

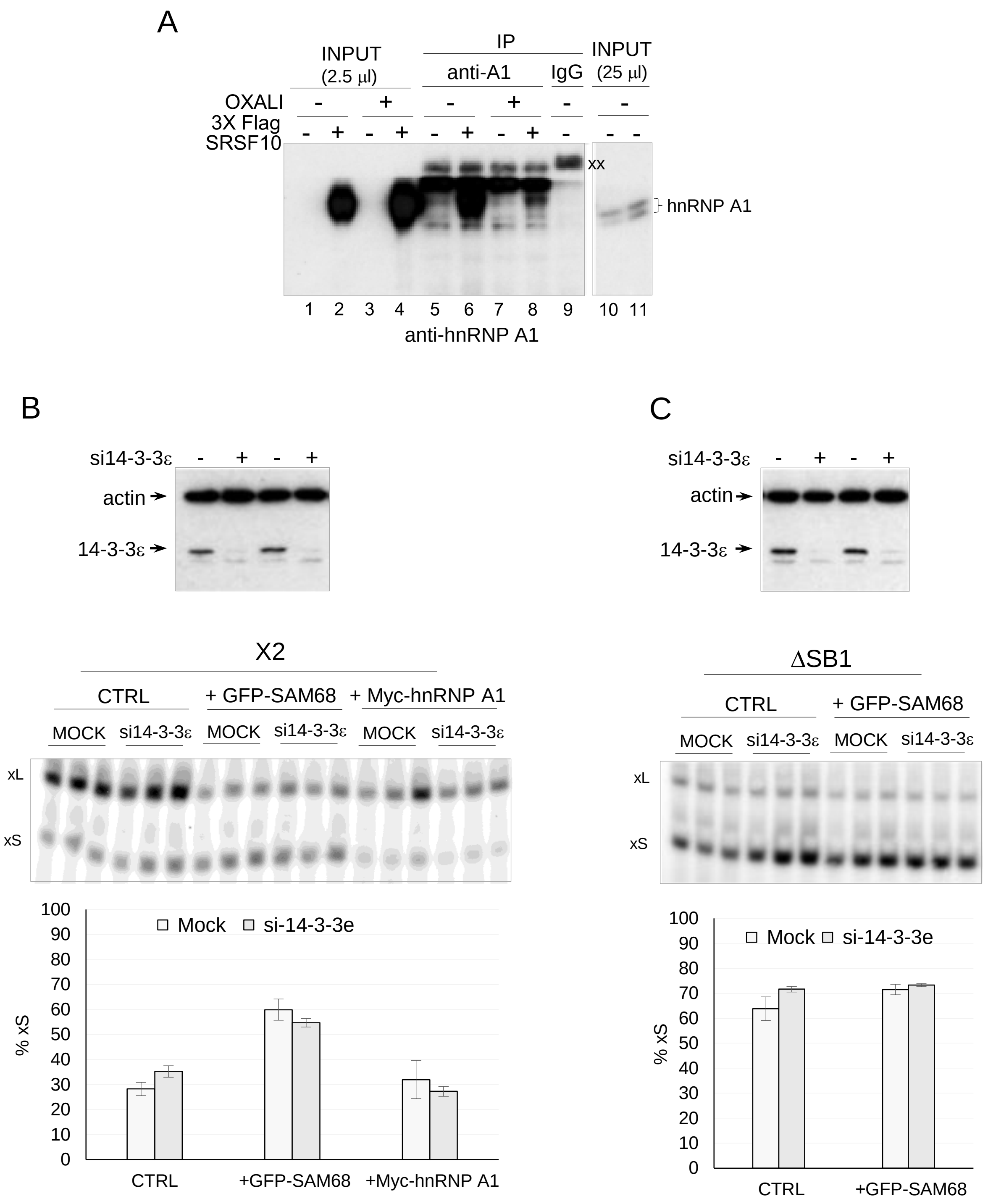

SUPPLEMENTARY FIGURE S3

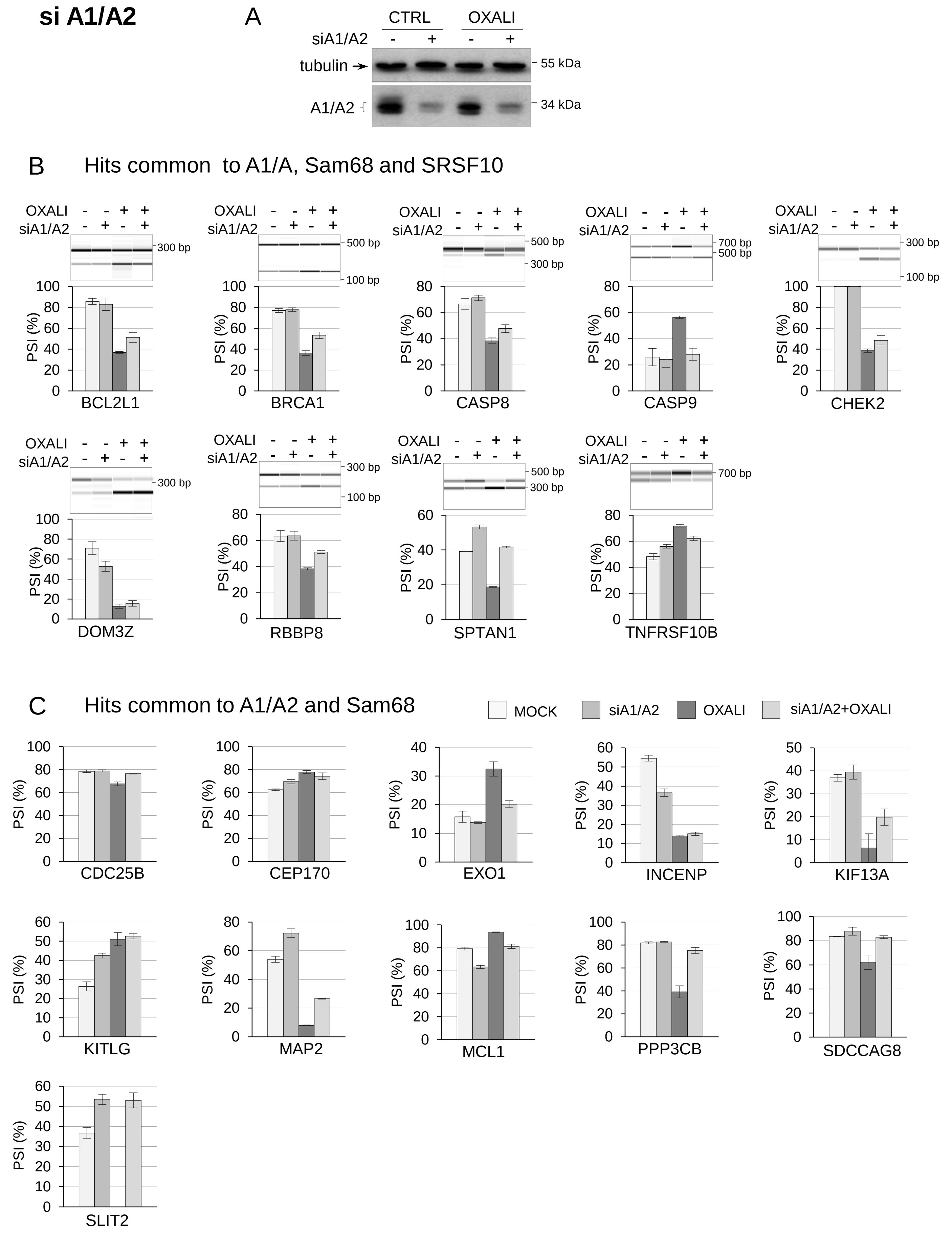

SUPPLEMENTARY FIGURE S4

si A1/A2

D Hits A1/A2

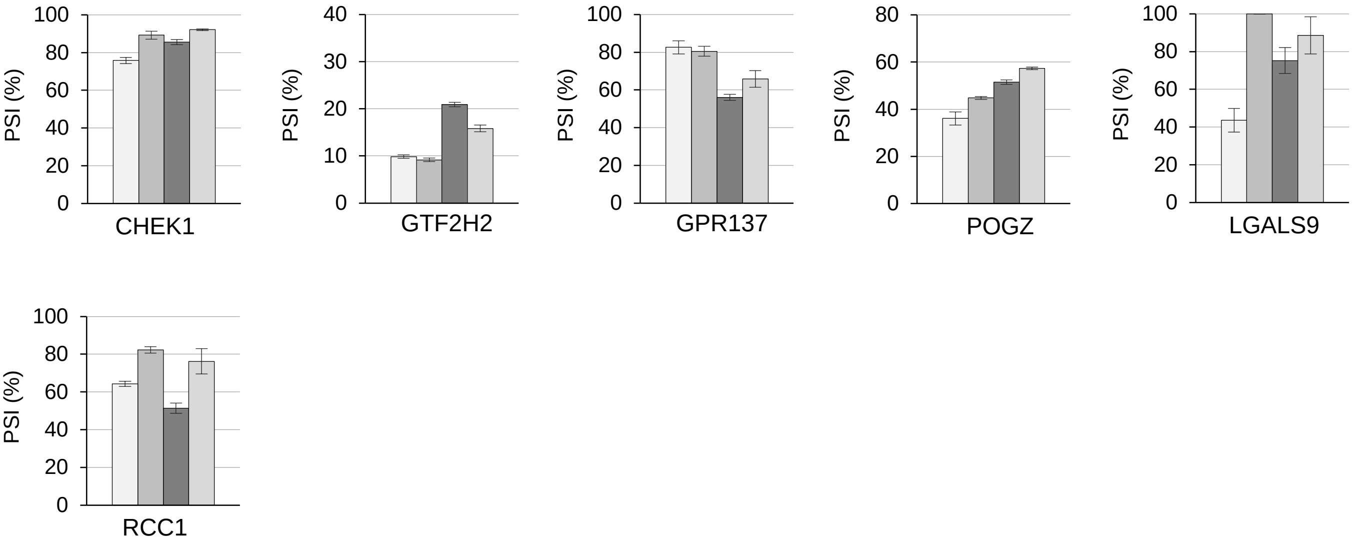

SUPPLEMENTARY FIGURE S4

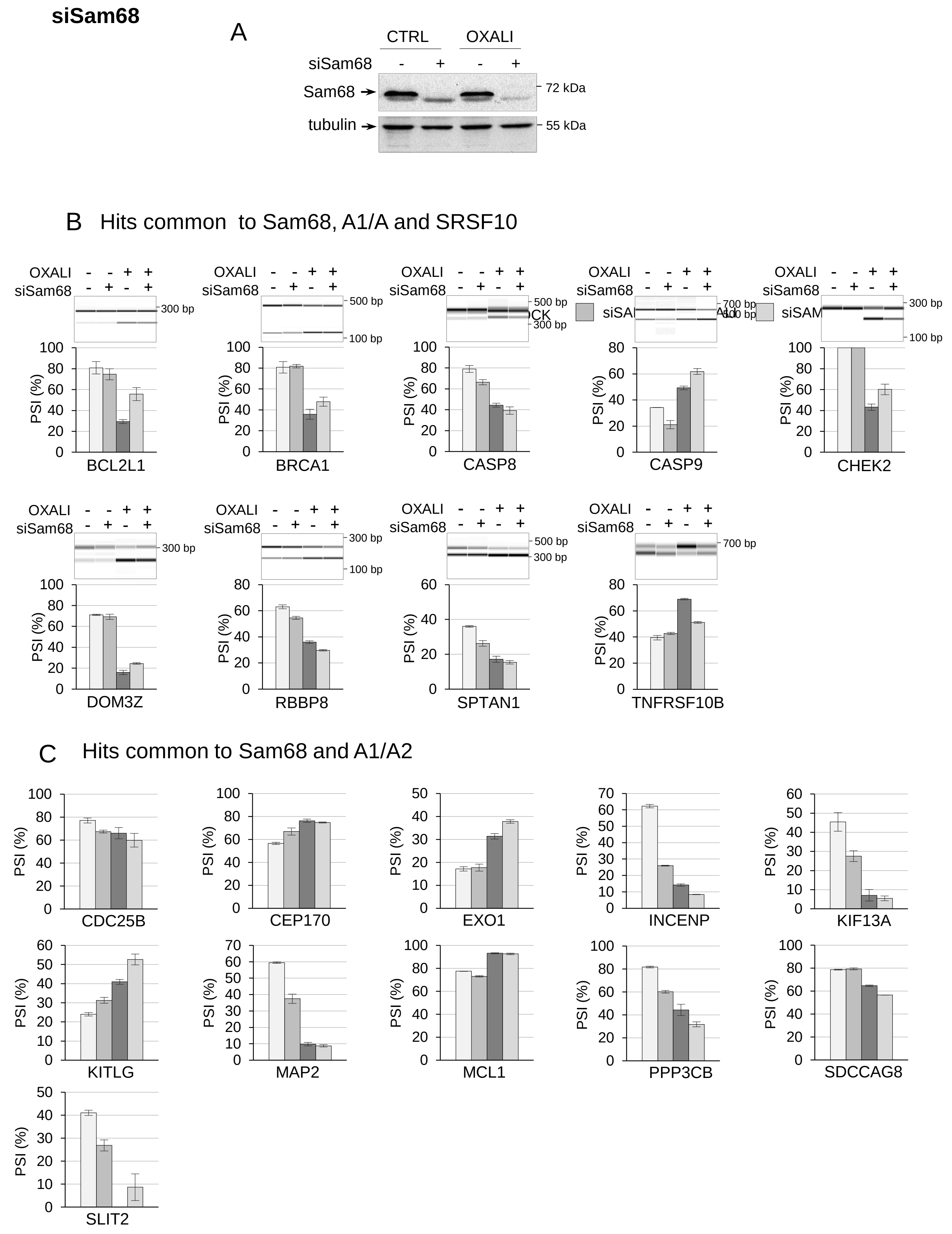

SUPPLEMENTARY FIGURE S5

siSam68

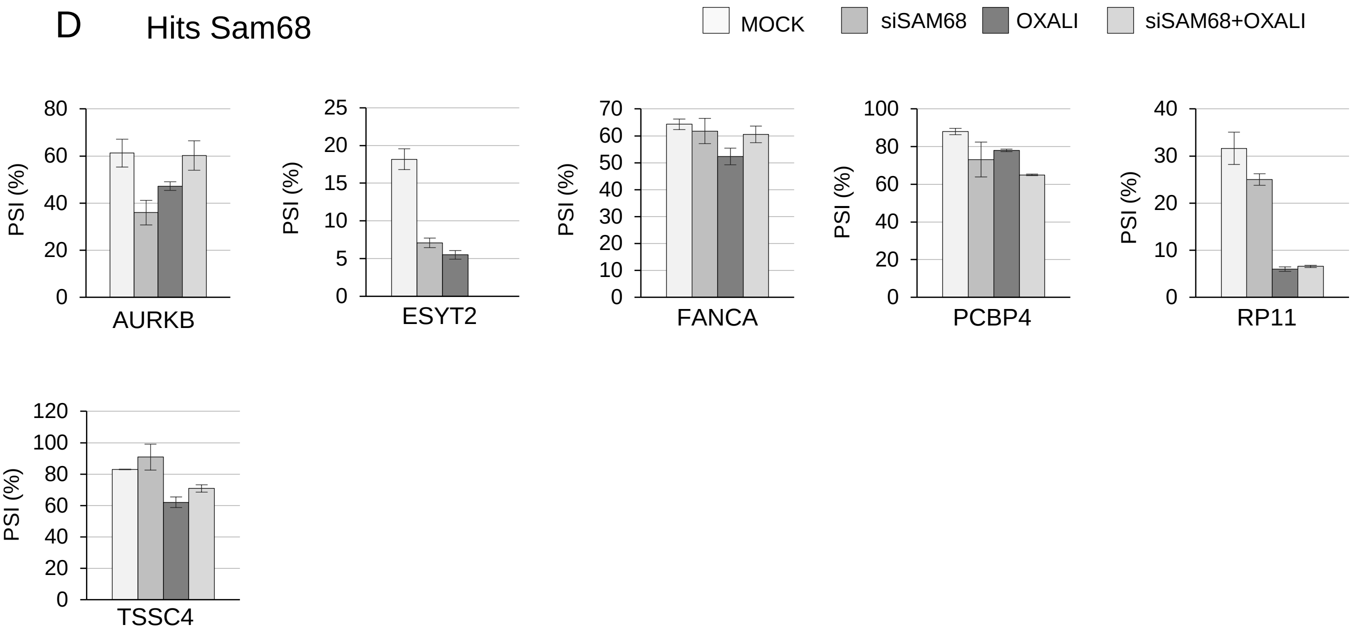

SUPPLEMENTARY FIGURE S5

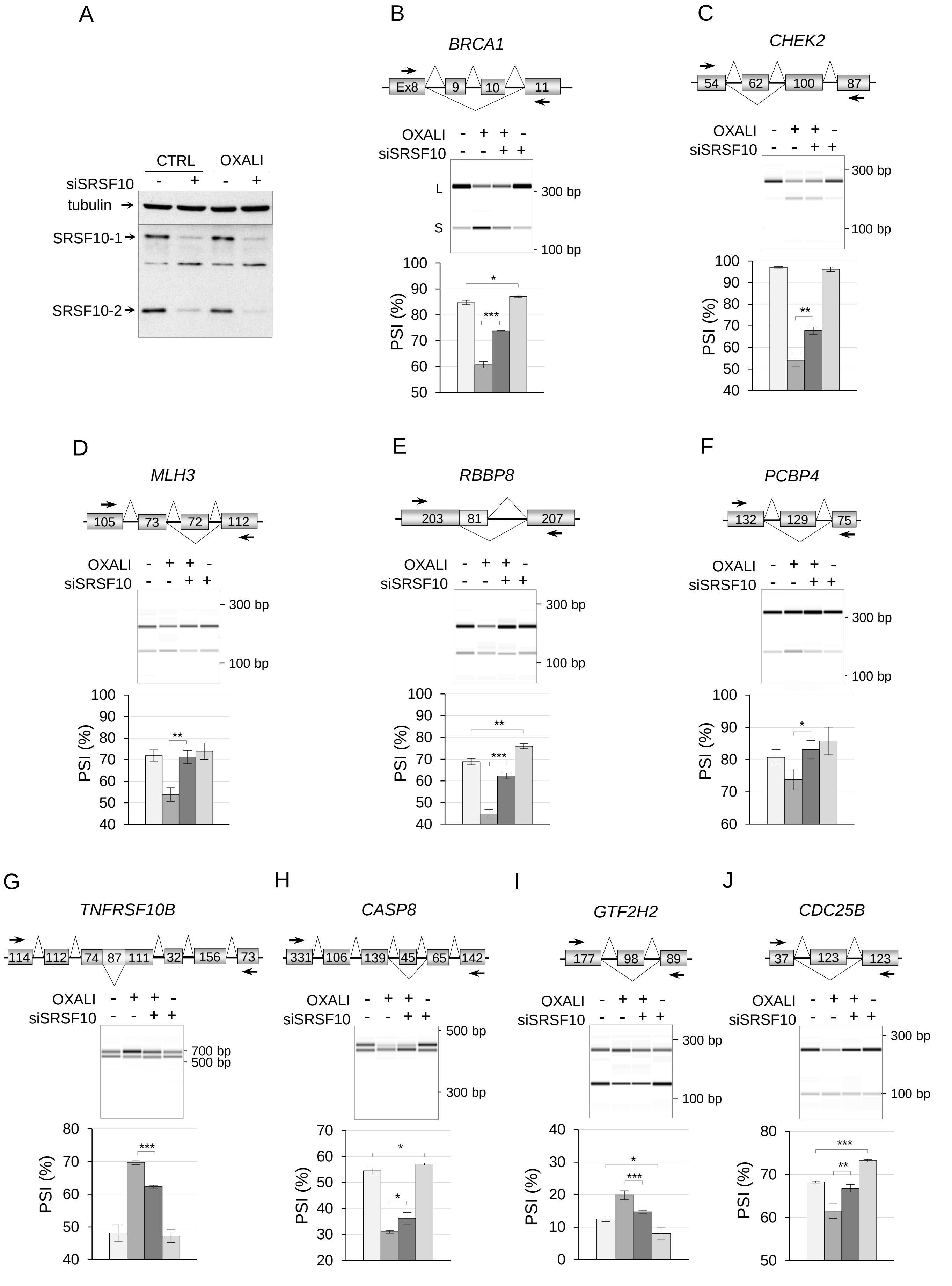

SUPPLEMENTARY FIGURE S6

SUPPLEMENTARY TABLE S1

Ct SYBR of RNA-IP Assays and **FOLD CHANGE** of BCL-X transcript quantities associated with hnRNP A1, Sam68, RNPS1, hnRNP F, hnRNP K, SRSF10 and hnRNP H proteins after oxaliplatin treatment .

IP-Assay # 1

Ct SYBR cDNA-Primer 3-1 REV, PCR-Primers 3-REV + 8-FWD

	CTRL	CTRL	CTRL	OXALIP	OXALIP	OXALIP
**INPUT-1**	22,73	23,16	22,66	23,13	23,31	23,32
**IP-hnRNP A1**	22,04	22,12	21,97	21,43	21,32	21,24
**IP-hnRNP K**	20,57	20,46	20,64	21,57	21,61	21,72
**IP-hnRNP H**	20,54	20,58	21,30	20,70	20,13	20,02
**FOLD CHANGE and st.dev**						
**hnRNP A1**	**2,20**	0,48				
**hnRNP K**	**0,64**	0,14				
**hnRNP H**	**2,02**	0,87				
**INPUT-2**	23,35	23,11	23,02	22,73	23,1	23,18
**IP-hnRNP F**	21,21	21,31	21,82	19,57	20,43	20,44
**IP-Sam68**	20,56	20,37	21,23	20,44	20,48	21,49
**FOLD CHANGE and st.dev**						
**hnRNP F**	**2,37**	1,06				
**Sam68**	**0,94**	0,50				
**INPUT-SRSF10**	23,34	23,31	23,20	22,8	23,53	23,15
**IP-SRSF10**	20,02	19,65	19,32	20,54	20,45	20,71
**FOLD CHANGE and st.dev**						
**SRSF10**	**0,51**	0,18				
**INPUT-Flag-RNPS1**	23,74	23,45	23,42	22,94	23,24	23,02
**IP-Flag-RNPS1**	20,79	19,96	19,73	20,51	20,66	20,39
**FOLD CHANGE and st.dev**						
**Flag-RNPS1**	**0,60**	0,25				
**INPUT-IgG**	24,87	25,36	25,3	25,41	25,04	25,23
**IP-IgG**	35,14	36,98	36,27	35,89	35,11	36,68
**FOLD CHANGE and st.dev**						
**IP-IgG**	**1,55**	1,26				

IP-Assay # 2

Ct SYBR cDNA-Primer 3-1-REV, PCR-Primers 3-REV + 8-FWD

	CTRL	CTRL	CTRL	OXALIP	OXALIP	OXALIP
**INPUT-1**	20,29	20,99	20,48	20,71	21,02	20,97
**IP-hnRNP A1**	19,28	19,49	19,46	18,86	19,12	18,92
**IP-hnRNP K**	17,94	17,29	17,26	18,93	18,74	18,74
**IP-hnRNP H**	18,79	19,08	18,58	18,22	17,95	17,80
**FOLD CHANGE and st.dev**						
**hnRNP A1**	**1,74**	0,52				
**hnRNP K**	**0,53**	0,20				
**hnRNP H**	**2,30**	0,81				
**INPUT-2**	20,24	20,85	20,68	20,81	20,84	20,73
**IP-hnRNP F**	18,72	18,85	18,62	17,68	18,23	17,31
**IP-Sam68**	18,19	18,25	18,14	18,43	18,55	18,44
**FOLD CHANGE and st.dev**						
**hnRNP F**	**2,41**	0,94				
**Sam68**	**0,96**	0,22				
**INPUT-SRSF10**	21,05	20,92	21,02	20,86	21,08	20,75
**IP-SRSF10**	17,47	17,47	17,16	18,05	17,78	18,1
**FOLD CHANGE and st.dev**						
**SRSF10**	**0,62**	0,13				
**INPUT-Flag-RNPS1**	21,36	21,13	20,88	20,54	20,71	20,64
**IP-Flag-RNPS1**	18,13	17,36	17,34	18,08	18,12	17,66
**FOLD CHANGE and st.dev**						
**Flag-RNPS1**	**0,59018**	0,23139				
**INPUT-IgG**	25,13	25,57	24,86	25,79	24,63	24,91
**IP-IgG**	35,49	36,36	36,63	35,69	36,58	35,42
**FOLD CHANGE and st.dev**						
**IP-IgG**	**1,31**	0,98				

IP-Assay # 3

Ct SYBR cDNA-Primer 3-1 REV, PCR-Primers 3-REV + 8-FWD

	CTRL	CTRL	CTRL	OXALIP	OXALIP	OXALIP
**INPUT-1**	20,06	20,38	20,06	20,33	20,54	20,51
**IP-hnRNP A1**	18,89	18,86	18,77	18,19	18,15	18,3
**IP-hnRNP K**	17,39	17,35	17,05	18,46	18,61	17,98
**IP-hnRNP H**	17,56	17,97	18,16	17,04	17,67	17,33
**FOLD CHANGE and st.dev**						
**hnRNP A1**	**1,91**	0,31				
**hnRNP K**	**0,59**	0,18				
**hnRNP H**	**1,86**	0,62				
**INPUT-2**	20,05	20,19	20,48	20,40	19,7	20,49
**IP-hnRNP F**	18,28	17,92	17,83	17,74	16,5	16,56
**IP-Sam68**	17,46	17,84	17,81	17,51	17,63	17,84
**FOLD CHANGE and st.dev**						
**hnRNP F**	**2,31**	1,35				
**Sam68**	**1,05**	0,40				
**INPUT-SRSF10**	20,35	20,54	20,35	20,04	20,44	20,42
**IP-SRSF10**	16,99	16,48	16,72	17,12	17,05	17,48
**FOLD CHANGE and st.dev**						
**SRSF10**	**0,68**	0,20				
**INPUT-Flag-RNPS1**	20,71	20,43	20,26	20,15	20,31	20,16
**IP-Flag-RNPS1**	17,06	16,62	16,48	17,57	17,43	16,96
**FOLD CHANGE and st.dev**						
**Flag-RNPS1**	**0,57**	0,20				
**INPUT-IgG**	23,45	23,08	23,12	22,98	23,52	23,38
**IP-IgG**	32,51	33,96	32,84	32,35	33,89	32,76
**IP-IgG**	**1,388**	1,031				

SUPPLEMENTARY TABLE S2

PSI-AVERAGE					St.dev					cut-off ΔPSI: > |10|, *P* value < 0.05				cut-off ΔPSI: >|5|, *P* value < 0.05				cut-off ΔPSI: >|5|, *P* value < 0.05			
Gene	CTRL	siA1/A2	OXA	OXA-siA1/A2	CTRL	siA1/A2	OXA	OXA-siA1/A2	Gene	ΔPSI(OXALI-CTRL)	st.dev	*P* values	Gene	ΔPSI(siA1/A2-CTRL)	st.dev	*P* values	Gene	ΔPSI(siA1/A2-OXALI)-(CTRL-OXALI)	st.dev	*P* values	
LGALS9	43,610	100,000	75,213	88,570	6,243	0,000	6,833	9,797	LGALS9	31,602	9,255	0,018528	LGALS9	56,390	6,243	0,002573	LGALS9	13,358	11,944	0,38228	reconf
CASP9	25,898	24,054	56,461	28,050	6,577	5,889	1,058	4,705	CASP9	30,564	6,661	0,008474	CASP9	-1,844	8,828	0,735816	CASP9	-28,412	4,823	0,00408	co-opt
MAPK10	70,246	68,509	100,000	100,000	6,565	3,564	0,000	0,000	MAPK10	29,754	6,565	0,008929	MAPK10	-1,738	7,470	0,707606	MAPK10	0,000	0,000	#DIV/0!	
KITLG	26,335	42,452	51,092	52,583	2,363	1,200	3,459	1,474	KITLG	24,757	4,189	0,000513	KITLG	16,117	2,650	0,00046	KITLG	1,491	3,760	0,52982	anti
TNFRSF10B	48,163	56,078	71,543	62,232	2,404	1,385	1,223	1,800	TNFRSF10B	23,379	2,697	0,000115	TNFRSF10B	7,914	2,774	0,007812	TNFRSF10B	-9,310	2,177	0,00177	reconf
F3	69,347	78,825	88,697	88,805	4,824	5,147	1,197	0,827	F3	19,351	4,971	0,002522	F3	9,478	7,054	0,080501	F3	0,108	1,455	0,90428	
EXO1	15,792	13,737	32,454	20,177	1,942	0,306	2,529	1,190	EXO1	16,663	3,189	0,000826	EXO1	-2,055	1,966	0,144562	EXO1	-12,277	2,795	0,00160	co-opt
CEP170	62,517	69,495	77,977	74,312	0,698	1,881	1,390	2,952	CEP170	15,460	1,555	0,000067	CEP170	6,978	2,007	0,003829	CEP170	-3,665	3,262	0,12358	anti
POGZ	36,134	44,791	51,467	57,383	2,796	0,638	0,982	0,567	POGZ	15,333	2,963	0,000858	POGZ	8,657	2,868	0,006391	POGZ	5,916	1,134	0,00083	same
MCL1	79,351	63,440	93,877	81,377	1,264	1,284	0,718	1,952	MCL1	14,525	1,454	0,000065	MCL1	-15,911	1,802	0,000107	MCL1	-12,499	2,080	0,00048	same
AURKA	7,160	11,991	19,098	17,525	3,356	3,786	0,984	1,391	AURKA	11,938	3,498	0,004099	AURKA	4,831	5,060	0,173504	AURKA	-1,573	1,704	0,18519	
GTF2H2	9,848	9,166	20,871	15,812	0,373	0,358	0,478	0,719	GTF2H2	11,024	0,606	0,000006	GTF2H2	-0,681	0,517	0,084607	GTF2H2	-5,059	0,863	0,00053	co-opt
CHEK1	75,816	89,218	85,586	92,141	1,674	2,172	1,326	0,422	CHEK1	9,770	2,136	0,001373	CHEK1	13,403	2,742	0,001067	CHEK1	6,555	1,391	0,00123	same
AURKB	48,173	35,887	56,310	37,592	5,441	3,272	5,370	5,611	AURKB	8,137	7,645	0,326121	AURKB	-12,286	6,349	0,068546	AURKB	-18,718	7,767	0,11678	
BCL2L11	42,471	53,834	49,422	63,933	1,636	5,958	1,197	5,074	BCL2L11	6,951	2,027	0,004031	BCL2L11	11,363	6,179	0,033367	BCL2L11	14,511	5,214	0,00852	
FOXM1	42,142	36,532	46,749	40,320	1,532	1,973	0,554	1,345	FOXM1	4,607	1,629	0,008056	FOXM1	-5,610	2,498	0,017699	FOXM1	-6,428	1,455	0,00157	
MLH3	58,751	62,053	59,355	69,408	3,016	5,432	1,413	3,173	MLH3	0,603	3,331	0,769438	MLH3	3,302	6,213	0,409427	MLH3	10,054	3,473	0,00742	
ARID4B	69,080	65,623	66,699	49,334	5,209	1,545	3,060	3,261	ARID4B	-2,382	6,041	0,715100	ARID4B	-3,458	5,433	0,463101	ARID4B	-17,365	4,472	0,04116	
AXIN1	20,347	15,384	17,243	25,478	1,217	13,360	2,101	2,897	AXIN1	-3,104	2,428	0,091257	AXIN1	-4,963	13,416	0,556532	AXIN1	8,234	3,579	0,01633	
BNIP1	3,570	0,000	0,000	0,000	6,184	0,000	0,000	0,000	BNIP1	-3,570	6,184	0,373901	BNIP1	-3,570	6,184	0,373901	BNIP1	0,000	0,000	#DIV/0!	
FASTK	78,968	66,095	74,578	69,149	2,937	2,407	2,387	6,902	FASTK	-4,391	3,785	0,159573	FASTK	-12,873	3,797	0,01224	FASTK	-5,429	7,303	0,26731	
CASC4	23,676	29,598	19,135	32,086	2,044	0,925	0,080	1,596	CASC4	-4,541	2,045	0,018375	CASC4	5,922	2,243	0,010243	CASC4	12,951	1,598	0,00015	
FANCA	58,004	62,350	51,981	52,145	1,062	0,881	1,191	1,890	FANCA	-6,024	1,596	0,002827	FANCA	4,345	1,380	0,005492	FANCA	0,164	2,234	0,90490	
BCLAF1	93,920	89,841	86,440	93,559	5,286	2,120	2,500	5,582	BCLAF1	-7,480	5,847	0,002969	BCLAF1	-4,079	5,695	0,060924	BCLAF1	7,120	6,116	0,01158	
AKIP1	41,131	38,003	32,354	34,174	3,855	1,405	1,721	2,730	AKIP1	-8,777	4,222	0,067415	AKIP1	-3,128	4,103	0,419141	AKIP1	1,820	3,227	0,38399	
BCLAF1	54,510	50,640	44,826	40,683	2,425	0,914	0,935	1,331	BCLAF1	-9,684	2,599	0,091035	BCLAF1	-3,870	2,592	0,282603	BCLAF1	-4,143	1,626	0,11398	
CDC25B	78,529	78,905	67,580	76,446	1,156	0,969	1,709	0,402	CDC25B	-10,949	2,063	0,000778	CDC25B	0,375	1,508	0,68862	CDC25B	8,866	1,755	0,00094	co-opt
ESYT2	16,894	14,592	5,342	5,470	0,376	1,235	0,337	0,383	ESYT2	-11,553	0,505	0,000002	ESYT2	-2,302	1,291	0,036619	ESYT2	0,129	0,510	0,68468	
UTRN	92,038	92,966	80,301	83,297	1,074	0,631	1,855	9,331	UTRN	-11,737	2,143	0,000690	UTRN	0,929	1,246	0,266151	UTRN	2,996	9,514	0,61446	
RCC1	64,335	82,313	51,399	76,241	1,334	1,650	2,657	6,725	RCC1	-12,936	2,974	0,001662	RCC1	17,978	2,122	0,000125	RCC1	24,842	7,231	0,00400	same
ZWINT	81,455	78,438	67,979	68,440	1,464	1,956	0,626	0,205	ZWINT	-13,476	1,592	0,000126	ZWINT	-3,018	2,443	0,099155	ZWINT	0,461	0,658	0,29178	
PCBP4	89,888	84,218	74,550	75,628	9,042	0,458	3,390	2,335	PCBP4	-15,337	9,657	0,051322	PCBP4	-5,670	9,054	0,339065	PCBP4	1,078	4,116	0,67368	
NAP1L4	26,779	22,737	10,623	12,102	1,454	1,975	0,183	0,490	NAP1L4	-16,157	1,466	0,000044	NAP1L4	-4,043	2,453	0,046177	NAP1L4	1,479	0,523	0,00804	
SPTAN1	39,097	53,250	18,730	41,677	0,000	1,576	0,260	0,499	SPTAN1	-20,367	0,260	0,000000	SPTAN1	14,153	1,576	1,25E-05	SPTAN1	22,947	0,562	0,00000	same
SDCCAG8	83,464	87,892	62,197	83,003	#DIV/0!	3,318	6,020	1,178	SDCCAG8	-21,267	#DIV/0!	#DIV/0!	SDCCAG8	4,429	#DIV/0!	#DIV/0!	SDCCAG8	20,806	6,134	0,04082	co-opt
TSSC4	79,365	78,241	58,081	57,229	7,823	3,346	2,607	3,830	TSSC4	-21,284	8,246	0,011068	TSSC4	-1,124	8,508	0,83017	TSSC4	-0,852	4,633	0,76612	
RP11	30,610	27,846	5,536	10,281	1,404	1,024	0,467	0,829	RP11	-25,075	1,480	0,000008	RP11	-2,764	1,738	0,051106	RP11	4,745	0,952	0,00099	
RBBP8	63,404	63,626	38,300	51,211	4,183	3,357	0,864	1,313	RBBP8	-25,104	4,272	0,000525	RBBP8	0,222	5,364	0,946235	RBBP8	12,911	1,572	0,00014	co-opt
GPR137	82,608	80,450	55,988	65,834	3,461	2,652	1,597	4,427	GPR137	-26,619	3,812	0,000268	GPR137	-2,157	4,361	0,439854	GPR137	9,845	4,707	0,02230	co-opt
CASP8	66,626	71,323	38,429	47,745	4,309	2,108	2,257	2,990	CASP8	-28,198	4,864	0,000553	CASP8	4,697	4,797	0,165129	CASP8	9,316	3,746	0,01258	co-opt
KIF13A	36,958	39,435	6,435	19,824	1,464	3,096	6,151	3,589	KIF13A	-30,523	6,323	0,001119	KIF13A	2,477	3,424	0,278423	KIF13A	13,389	7,121	0,03119	co-opt
SLIT2	36,714	53,495	0,000	52,980	2,835	2,542	0,000	3,766	SLIT2	-36,714	2,835	0,000023	SLIT2	16,781	3,808	0,001583	SLIT2	52,980	3,766	0,00002	co-opt
INCENP	54,581	36,581	13,878	15,114	1,534	2,003	0,518	0,847	INCENP	-40,703	1,619	0,000002	INCENP	-18,001	2,523	0,001388	INCENP	1,235	0,993	0,09737	anti
BRCA1	77,050	77,716	36,309	53,289	1,860	1,870	2,316	3,098	BRCA1	-40,740	2,971	0,000019	BRCA1	0,667	2,638	0,684097	BRCA1	16,980	3,868	0,00161	co-opt
PPP3CB	81,948	82,689	39,221	75,239	0,949	0,589	5,240	2,738	PPP3CB	-42,728	5,326	0,000156	PPP3CB	0,741	1,117	0,314799	PPP3CB	36,018	5,912	0,00046	co-opt
MAP2	54,002	72,290	8,031	26,518	2,116	2,959	0,273	0,317	MAP2	-45,971	2,134	0,000003	MAP2	18,288	3,638	0,003736	MAP2	18,488	0,418	0,00001	same
BCL2L1	85,723	83,062	36,806	51,171	2,840	5,986	1,073	4,590	BCL2L1	-48,917	3,036	0,000092	BCL2L1	-2,661	6,625	0,611354	BCL2L1	14,364	4,714	0,00618	co-opt
DOM3Z	71,019	52,746	12,718	15,606	6,505	5,080	2,167	2,791	DOM3Z	-58,300	6,856	0,000124	DOM3Z	-18,273	8,253	0,018541	DOM3Z	2,887	3,534	0,22997	anti
CHEK2	100,000	100,000	38,574	48,420	0,000	0,000	1,595	4,417	CHEK2	-61,426	1,595	0,000000	CHEK2	0,000	0,000	#DIV/0!	CHEK2	9,846	4,696	0,02213	co-opt

SUPPLEMENTARY TABLE S3

PSI-AVERAGE					St.dev			
**Gene**	**CTRL**	**siSAM68**	**OXA**	**OXA-siSAM68**	**CTRL**	**siSAM68**	**OXA**	**OXA-siSAM68**
**LGALS9**	**29,748**	**55,538**	**66,252**	**94,387**	24,330	19,928	9,984	9,722
**TNFRSF10B**	**39,519**	**42,733**	**69,019**	**51,231**	1,781	0,887	0,555	0,701
**POGZ**	**31,186**	**28,878**	**52,763**	**47,133**	3,253	1,370	1,438	3,707
**CEP170**	**56,504**	**66,836**	**76,201**	**74,675**	2,186	2,154	0,711	1,125
**KITLG**	**24,014**	**31,294**	**40,968**	**52,648**	0,929	1,486	1,301	2,869
**F3**	**69,338**	**65,017**	**85,202**	**77,709**	8,555	5,600	2,585	4,630
**MCL1**	**77,466**	**73,088**	**93,196**	**92,617**	0,073	0,594	0,487	0,645
**CASP9**	**34,253**	**21,132**	**49,273**	**61,878**	2,143	1,255	4,957	5,951
**AURKA**	**5,846**	**5,536**	**20,686**	**18,826**	5,128	0,807	1,444	0,663
**EXO1**	**17,132**	**17,678**	**31,363**	**37,821**	0,958	1,525	1,178	0,753
**BCL2L11**	**39,097**	**36,762**	**53,293**	**58,571**	2,252	5,346	3,417	0,280
**GTF2H2**	**8,079**	**7,591**	**19,169**	**18,593**	0,391	0,930	0,952	0,906
**CHEK1**	**79,399**	**78,521**	**83,517**	**83,130**	2,552	1,926	1,927	1,293
**FOXM1**	**40,999**	**26,567**	**44,259**	**36,414**	0,652	1,234	1,233	2,150
**CDC25A**	**87,432**	**59,642**	**86,448**	**59,458**	21,769	1,213	23,472	0,915
**AXIN1**	**20,128**	**21,242**	**18,447**	**20,940**	4,281	6,885	1,497	1,720
**BCLAF1**	**48,031**	**48,599**	**44,908**	**47,195**	1,507	0,353	1,396	1,265
**BCLAF1**	**92,681**	**90,124**	**87,434**	**83,356**	6,357	2,775	1,723	0,523
**CASC4**	**24,519**	**23,558**	**18,695**	**17,187**	0,933	0,768	0,635	0,772
**AKIP1**	**42,796**	**49,510**	**34,640**	**37,691**	1,372	3,695	2,385	3,545
**ARID4B**	**73,053**	**75,063**	**64,275**	**71,380**	4,199	1,921	9,967	1,204
**FASTK**	**75,403**	**73,020**	**65,992**	**76,719**	2,395	3,097	8,165	4,300
**RCC1**	**67,671**	**69,407**	**58,210**	**48,261**	2,756	1,500	3,192	5,418
**PCBP4**	**88,006**	**73,103**	**78,019**	**64,981**	1,743	9,267	0,623	0,472
**CDC25B**	**77,104**	**67,465**	**66,087**	**59,852**	0,962	3,171	1,388	0,496
**MLH3**	**72,984**	**68,761**	**61,548**	**63,156**	8,233	8,149	2,664	6,709
**MAPK10**	**100,000**	**100,000**	**88,355**	**100,000**	0,000	0,000	20,169	0,000
**FANCA**	**64,308**	**61,767**	**52,325**	**60,579**	1,936	4,718	3,098	3,088
**ESYT2**	**18,165**	**7,084**	**5,492**	**0,000**	1,384	0,641	0,562	0,000
**SDCCAG8**	**78,741**	**79,382**	**64,704**	**56,638**	0,373	0,839	0,655	#DIV/0!
**AURKB**	**61,268**	**35,970**	**47,230**	**60,200**	5,932	5,291	1,854	8,249
**ZWINT**	**79,628**	**79,026**	**65,142**	**64,714**	1,865	2,135	1,640	2,259
**UTRN**	**94,194**	**91,464**	**75,987**	**84,032**	5,151	0,502	5,688	6,655
**SPTAN1**	**36,006**	**26,241**	**17,154**	**15,375**	0,431	1,659	0,000	0,000
**NAP1L4**	**29,925**	**27,106**	**11,051**	**12,184**	0,545	3,282	1,361	2,865
**TSSC4**	**82,962**	**90,848**	**62,080**	**70,935**	0,181	8,326	3,381	2,329
**RP11**	**31,621**	**25,013**	**5,994**	**6,581**	3,440	1,203	0,459	0,218
**GPR137**	**82,295**	**78,633**	**56,138**	**54,873**	7,451	6,932	5,941	3,107
**RBBP8**	**63,007**	**54,510**	**35,963**	**29,693**	1,446	1,204	1,046	0,551
**CASP8**	**79,049**	**66,312**	**44,383**	**39,172**	0,069	3,219	1,398	2,267
**PPP3CB**	**81,599**	**60,181**	**44,338**	**31,721**	0,749	1,153	4,898	2,213
**KIF13A**	**45,458**	**27,584**	**7,083**	**5,495**	4,837	2,829	3,028	1,229
**SLIT2**	**41,083**	**26,883**	**0,000**	**8,692**	1,157	2,435	0,000	7,800
**BRCA1**	**80,787**	**81,833**	**35,867**	**47,852**	3,330	2,496	1,933	3,479
**INCENP**	**62,232**	**25,957**	**14,198**	**8,401**	1,062	0,307	0,732	0,162
**MAP2**	**59,500**	**37,482**	**9,776**	**8,735**	0,436	2,862	1,056	0,711
**BCL2L1**	**80,840**	**74,719**	**29,411**	**55,723**	5,463	1,717	4,740	4,399
**DOM3Z**	**71,169**	**69,151**	**15,868**	**24,522**	0,623	2,512	2,087	0,713
**CHEK2**	**100,000**	**100,000**	**43,188**	**60,176**	0,000	0,000	2,985	5,086

	cut-off ΔPSI: > |10|, *P* value < 0.05				cut-off ΔPSI: >|5|, *P* value < 0.05				cut-off ΔPSI: >|5|, *P* value < 0.05			
**Gene**	**ΔPSI(OXALI-CTRL)**	st.dev	*P* values	**Gene**	**ΔPSI(siSAM68-CTRL)**	st.dev	*P* values	**Gene**	**ΔPSI(siSAM68-OXALI)-(CTRL-OXALI)**	st.dev	*P* values	
**LGALS9**	**36,505**	24,094	0,224893	**LGALS9**	**25,790**	31,156	0,440022	**LGALS9**	**28,135**	13,521	0,197008	
**TNFRSF10B**	**29,500**	1,865	0,000011	**TNFRSF10B**	**3,215**	1,989	0,048867	**TNFRSF10B**	**-17,787**	0,894	0,000004	co-opt
**POGZ**	**21,577**	3,557	0,000464	**POGZ**	**-2,308**	3,530	0,320680	**POGZ**	**-5,630**	3,976	0,070229	
**CEP170**	**19,697**	2,298	0,000120	**CEP170**	**10,331**	3,069	0,004310	**CEP170**	**-1,526**	1,331	0,118002	anti
**KITLG**	**16,953**	1,599	0,000052	**KITLG**	**7,280**	1,753	0,001979	**KITLG**	**11,681**	3,151	0,003023	same
**F3**	**15,864**	8,937	0,037132	**F3**	**-4,321**	10,225	0,504750	**F3**	**-7,493**	5,302	0,070629	
**MCL1**	**15,731**	0,493	0,000001	**MCL1**	**-4,378**	0,598	0,000223	**MCL1**	**-0,580**	0,808	0,282180	anti
**CASP9**	**15,020**	5,401	0,008542	**CASP9**	**-13,122**	2,484	0,000792	**CASP9**	**12,604**	7,745	0,047894	reconf
**AURKA**	**14,840**	5,327	0,008493	**AURKA**	**-0,310**	5,191	0,922534	**AURKA**	**-1,860**	1,589	0,112496	
**EXO1**	**14,231**	1,518	0,000084	**EXO1**	**0,547**	1,801	0,626727	**EXO1**	**6,459**	1,398	0,001322	co-opt
**BCL2L11**	**14,196**	4,092	0,003862	**BCL2L11**	**-2,335**	5,802	0,524107	**BCL2L11**	**5,278**	3,428	0,056004	
**GTF2H2**	**11,090**	1,029	0,000048	**GTF2H2**	**-0,488**	1,009	0,449243	**GTF2H2**	**-0,576**	1,314	0,489912	
**CHEK1**	**4,118**	3,198	0,089563	**CHEK1**	**-0,878**	3,197	0,659207	**CHEK1**	**-0,387**	2,321	0,787287	
**FOXM1**	**3,260**	1,395	0,015508	**FOXM1**	**-14,432**	1,396	0,000057	**FOXM1**	**-7,845**	2,478	0,005389	
**CDC25A**	**-0,984**	32,013	0,960112	**CDC25A**	**-27,789**	21,803	0,091865	**CDC25A**	**-26,990**	23,490	0,220717	
**AXIN1**	**-1,681**	4,535	0,652481	**AXIN1**	**1,114**	8,108	0,855194	**AXIN1**	**2,493**	2,280	0,196261	
**BCLAF1**	**-3,124**	2,054	0,057892	**BCLAF1**	**0,567**	1,547	0,560013	**BCLAF1**	**2,287**	1,883	0,103259	
**BCLAF1**	**-5,248**	6,586	0,239655	**BCLAF1**	**-2,558**	6,936	0,557749	**BCLAF1**	**-4,078**	1,800	0,017205	
**CASC4**	**-5,825**	1,129	0,000866	**CASC4**	**-0,961**	1,208	0,240338	**CASC4**	**-1,507**	0,999	0,059262	
**AKIP1**	**-8,156**	2,752	0,006819	**AKIP1**	**6,715**	3,941	0,041931	**AKIP1**	**3,051**	4,273	0,283777	
**ARID4B**	**-8,778**	10,815	0,232475	**ARID4B**	**2,010**	4,617	0,492869	**ARID4B**	**7,105**	10,039	0,287485	
**FASTK**	**-9,411**	8,509	0,127906	**FASTK**	**-2,383**	3,915	0,351260	**FASTK**	**10,728**	9,228	0,114346	
**RCC1**	**-9,461**	4,217	0,017756	**RCC1**	**1,736**	3,137	0,392024	**RCC1**	**-9,949**	6,289	0,051885	
**PCBP4**	**-9,986**	1,851	0,000731	**PCBP4**	**-14,903**	9,429	0,052039	**PCBP4**	**-13,038**	0,782	0,000009	co-opt
**CDC25B**	**-11,017**	1,688	0,000349	**CDC25B**	**-9,639**	3,313	0,007288	**CDC25B**	**-6,235**	1,474	0,001846	same
**MLH3**	**-11,436**	8,653	0,083925	**MLH3**	**-4,222**	11,583	0,562074	**MLH3**	**1,608**	7,219	0,719229	
**MAPK10**	**-11,645**	20,169	0,373901	**MAPK10**	**0,000**	0,000	#DIV/0!	**MAPK10**	**11,645**	20,169	0,495025	
**FANCA**	**-11,983**	3,653	0,004737	**FANCA**	**-2,541**	5,100	0,436909	**FANCA**	**8,253**	4,374	0,030843	co-opt
**ESYT2**	**-12,673**	1,494	0,000125	**ESYT2**	**-11,081**	1,525	0,000229	**ESYT2**	**-5,492**	0,562	0,000072	same
**SDCCAG8**	**-14,037**	0,754	0,001439	**SDCCAG8**	**0,640**	0,918	0,400537	**SDCCAG8**	**-8,067**	#DIV/0!	#DIV/0!	co-opt
**AURKB**	**-14,037**	6,215	0,053289	**AURKB**	**-25,298**	7,949	0,016828	**AURKB**	**12,970**	8,455	0,128597	reconf
**ZWINT**	**-14,486**	2,484	0,000540	**ZWINT**	**-0,603**	2,835	0,731425	**ZWINT**	**-0,428**	2,792	0,803589	
**UTRN**	**-18,207**	7,674	0,014741	**UTRN**	**-2,730**	5,176	0,412558	**UTRN**	**8,045**	8,755	0,186688	
**SPTAN1**	**-18,852**	0,431	0,000000	**SPTAN1**	**-9,765**	1,714	0,000592	**SPTAN1**	**-1,779**	0,000	#DIV/0!	anti
**NAP1L4**	**-18,874**	1,466	0,000024	**NAP1L4**	**-2,818**	3,327	0,216237	**NAP1L4**	**1,133**	3,171	0,569589	
**TSSC4**	**-20,881**	3,385	0,003688	**TSSC4**	**7,887**	8,328	0,293419	**TSSC4**	**8,854**	4,105	0,020192	co-opt
**RP11**	**-25,627**	3,471	0,000215	**RP11**	**-6,608**	3,645	0,034840	**RP11**	**0,587**	0,508	0,116213	anti
**GPR137**	**-26,157**	9,530	0,021510	**GPR137**	**-3,662**	10,177	0,612009	**GPR137**	**-1,265**	6,705	0,760210	
**RBBP8**	**-27,044**	1,785	0,000013	**RBBP8**	**-8,497**	1,882	0,001442	**RBBP8**	**-6,270**	1,182	0,000780	same
**CASP8**	**-34,666**	1,400	0,000060	**CASP8**	**-12,736**	3,219	0,013054	**CASP8**	**-5,211**	2,664	0,027573	same
**PPP3CB**	**-37,261**	4,955	0,000201	**PPP3CB**	**-21,418**	1,374	0,000011	**PPP3CB**	**-12,617**	5,375	0,015276	same
**KIF13A**	**-38,376**	5,707	0,000311	**KIF13A**	**-17,874**	5,604	0,005242	**KIF13A**	**-1,588**	3,268	0,447372	anti
**SLIT2**	**-41,083**	1,157	0,000000	**SLIT2**	**-14,200**	2,695	0,000800	**SLIT2**	**8,692**	7,800	0,125787	anti
**BRCA1**	**-44,921**	3,851	0,000035	**BRCA1**	**1,046**	4,162	0,685887	**BRCA1**	**11,985**	3,980	0,006446	co-opt
**INCENP**	**-48,034**	1,290	0,000000	**INCENP**	**-36,275**	1,106	0,000001	**INCENP**	**-5,797**	0,750	0,000180	anti
**MAP2**	**-49,723**	1,143	0,000010	**MAP2**	**-22,018**	2,895	0,001972	**MAP2**	**-1,042**	1,273	0,229436	anti
**BCL2L1**	**-51,429**	7,233	0,000250	**BCL2L1**	**-6,121**	5,727	0,137781	**BCL2L1**	**26,311**	6,466	0,002137	co-opt
**DOM3Z**	**-55,302**	2,178	0,000002	**DOM3Z**	**-2,018**	2,588	0,248199	**DOM3Z**	**8,654**	2,205	0,002448	co-opt
**CHEK2**	**-56,812**	2,985	0,000005	**CHEK2**	**0,000**	0,000	#DIV/0!	**CHEK2**	**16,989**	5,897	0,007543	co-opt

SUPPLEMENTARY TABLE S4

PSI-AVERAGE					St.dev					cut-off ΔPSI: > |10|				cut-off ΔPSI: >|5|, *P* value < 0.05				cut-off ΔPSI: >|5|, *P* value < 0.05			
**Gene**	**CTRL**	**siSRSF10**	**OXA**	**OXA-siSRSF10**	**CTRL**	**siSRSF10**	**OXA**	**OXA-siSRSF10**	**Gene**	**ΔPSI(OXALI-CTRL)**	st.dev	*P* values	**Gene**	**ΔPSI(siSRSF10-CTRL)**	st.dev	*P* values	**Gene**	**ΔPSI(siSRSF10-OXALI)-(CTRL-OXALI)**	st.dev	*P* values	
**TNFRSF10B**	**48,168**	**47,178**	**69,773**	**62,300**	2,526	1,935	0,706	0,388	**TNFRSF10B**	**21,604**	2,623	0,000140	**TNFRSF10B**	**-0,990**	3,182	0,618470	**TNFRSF10B**	**-7,473**	0,8054	0,000088	co-opt
**CASP9**	**40,734**	**55,601**	**55,243**	**69,129**	5,082	5,489	4,400	6,728	**CASP9**	**14,509**	6,722	0,020143	**CASP9**	**14,867**	7,480	0,026238	**CASP9**	**13,886**	8,0392	0,040269	same
**MCL1**	**85,801**	**81,784**	**94,283**	**90,820**	1,058	1,463	0,191	0,601	**MCL1**	**8,482**	1,075	0,000166	**MCL1**	**-4,017**	1,806	0,018252	**MCL1**	**-3,463**	0,6302	0,000681	
**GTF2H2**	**12,524**	**8,027**	**19,852**	**14,725**	0,816	1,938	1,314	0,481	**GTF2H2**	**7,328**	1,547	0,001201	**GTF2H2**	**-4,497**	2,102	0,020752	**GTF2H2**	**-5,128**	1,3989	0,003153	
**AURKA**	**9,370**	**7,784**	**16,332**	**14,419**	1,053	1,157	0,526	0,293	**AURKA**	**6,962**	1,177	0,000512	**AURKA**	**-1,586**	1,565	0,154059	**AURKA**	**-1,913**	0,6024	0,005321	
**AURKB**	**54,012**	**59,842**	**60,633**	**69,988**	4,398	4,130	5,516	6,726	**AURKB**	**6,622**	7,054	0,179320	**AURKB**	**5,831**	6,033	0,169429	**AURKB**	**9,355**	8,6985	0,135968	
**EXO1**	**18,726**	**16,991**	**24,585**	**27,020**	0,555	1,141	1,109	0,550	**EXO1**	**5,859**	1,241	0,001216	**EXO1**	**-1,735**	1,269	0,077017	**EXO1**	**2,435**	1,2382	0,027126	
**CHEK1**	**77,992**	**86,924**	**83,220**	**92,676**	1,553	1,528	1,684	0,315	**CHEK1**	**5,229**	2,291	0,016776	**CHEK1**	**8,933**	2,179	0,002077	**CHEK1**	**9,456**	1,7136	0,000669	
**BAX**	**91,223**	**92,019**	**96,129**	**96,726**	2,082	1,791	1,062	1,323	**BAX**	**4,906**	2,337	0,022047	**BAX**	**0,796**	2,746	0,642049	**BAX**	**0,597**	1,6971	0,574947	
**CDKN1A**	**0,000**	**0,000**	**0,323**	**1,009**	0,000	0,000	0,559	0,111	**CDKN1A**	**0,323**	0,559	0,373901	**CDKN1A**	**0,000**	0,000	#DIV/0!	**CDKN1A**	**0,686**	0,5703	0,105781	
**AKAP13**	**0,475**	**0,890**	**0,796**	**0,972**	0,478	0,428	0,134	0,017	**AKAP13**	**0,320**	0,497	0,326775	**AKAP13**	**0,414**	0,642	0,326214	**AKAP13**	**0,176**	0,1348	0,086761	
**CDK1**	**97,514**	**97,345**	**97,277**	**98,065**	0,559	0,195	0,373	0,137	**CDK1**	**-0,237**	0,672	0,574070	**CDK1**	**-0,168**	0,592	0,647727	**CDK1**	**0,789**	0,397	0,026265	
**CDK2**	**98,582**	**98,185**	**97,962**	**98,069**	0,443	0,156	0,155	0,062	**CDK2**	**-0,620**	0,469	0,084037	**CDK2**	**-0,397**	0,470	0,216825	**CDK2**	**0,107**	0,1668	0,330514	
**BCLAF1**	**59,925**	**2,519**	**58,282**	**1,410**	3,635	1,188	12,012	1,342	**BCLAF1**	**-1,643**	12,550	0,831718	**BCLAF1**	**-57,406**	3,824	0,000013	**BCLAF1**	**-56,871**	12,087	0,001234	
**ITGB3BP**	**3,456**	**20,958**	**0,000**	**31,695**	5,986	1,026	0,000	1,920	**ITGB3BP**	**-3,456**	5,986	0,373901	**ITGB3BP**	**17,502**	6,074	0,007538	**ITGB3BP**	**31,695**	1,9203	0,000009	
**CHEK2-2**	**97,303**	**97,118**	**93,620**	**95,814**	0,261	0,097	1,756	0,520	**CHEK2-2**	**-3,683**	1,775	0,022889	**CHEK2-2**	**-0,185**	0,278	0,313575	**CHEK2-2**	**2,194**	1,8312	0,106655	
**RAD17**	**95,538**	**93,442**	**91,448**	**91,386**	1,333	0,315	0,716	1,458	**RAD17**	**-4,090**	1,513	0,009443	**RAD17**	**-2,095**	1,370	0,057055	**RAD17**	**-0,062**	1,6247	0,950845	
**APAF1**	**62,624**	**55,931**	**58,116**	**54,992**	7,779	4,280	2,697	3,411	**APAF1**	**-4,508**	8,234	0,396638	**APAF1**	**-6,693**	8,879	0,261705	**APAF1**	**-3,123**	4,3485	0,281411	
**CDC25A**	**63,721**	**63,035**	**58,607**	**61,623**	1,054	0,520	1,516	0,670	**CDC25A**	**-5,114**	1,846	0,008665	**CDC25A**	**-0,686**	1,175	0,369193	**CDC25A**	**3,016**	1,6574	0,034442	
**AXIN1**	**17,509**	**19,955**	**11,689**	**12,881**	1,142	4,447	2,158	2,122	**AXIN1**	**-5,820**	2,441	0,014503	**AXIN1**	**2,446**	4,591	0,408353	**AXIN1**	**1,192**	3,0264	0,532554	
**FASTK**	**84,327**	**88,941**	**77,901**	**77,839**	0,218	0,842	1,340	1,775	**FASTK**	**-6,427**	1,358	0,001205	**FASTK**	**4,614**	0,870	0,000779	**FASTK**	**-0,062**	2,2241	0,964097	
**CDC25B**	**68,219**	**73,237**	**61,499**	**66,785**	0,232	0,301	1,716	0,908	**CDC25B**	**-6,719**	1,732	0,002552	**CDC25B**	**5,019**	0,380	0,000022	**CDC25B**	**5,286**	1,9416	0,009204	
**CASC4**	**27,677**	**30,583**	**20,571**	**23,975**	1,525	1,026	0,695	1,050	**CASC4**	**-7,106**	1,676	0,001832	**CASC4**	**2,906**	1,839	0,052043	**CASC4**	**3,403**	1,2589	0,009430	
**AKIP1**	**31,375**	**36,311**	**23,859**	**28,001**	1,392	1,810	1,266	1,861	**AKIP1**	**-7,516**	1,882	0,002292	**AKIP1**	**4,936**	2,283	0,020043	**AKIP1**	**4,142**	2,2505	0,033289	
**ARID4B**	**21,731**	**11,572**	**14,028**	**18,836**	0,428	4,150	2,484	1,327	**ARID4B**	**-7,703**	2,520	0,025744	**ARID4B**	**-10,160**	4,172	0,046573	**ARID4B**	**4,809**	2,8161	0,041656	
**PCBP4**	**80,686**	**85,773**	**72,848**	**83,086**	2,419	4,238	2,347	2,855	**PCBP4**	**-7,837**	3,370	0,015756	**PCBP4**	**5,088**	4,879	0,145229	**PCBP4**	**10,237**	3,6954	0,008659	
**NAP1L4**	**20,797**	**27,897**	**6,892**	**15,675**	1,019	2,748	0,584	1,406	**NAP1L4**	**-13,904**	1,174	0,000033	**NAP1L4**	**7,100**	2,931	0,013736	**NAP1L4**	**8,783**	1,5223	0,000563	same
**ESYT2**	**24,689**	**23,736**	**9,951**	**12,114**	0,845	1,627	1,856	1,073	**ESYT2**	**-14,738**	2,039	0,000234	**ESYT2**	**-0,953**	1,834	0,419078	**ESYT2**	**2,163**	2,1439	0,155443	
**SPTAN1**	**25,769**	**30,304**	**10,311**	**19,557**	1,068	0,922	1,233	2,426	**SPTAN1**	**-15,458**	1,631	0,000081	**SPTAN1**	**4,534**	1,410	0,005097	**SPTAN1**	**9,245**	2,7219	0,004172	co-opt
**BCLAF1**	**49,333**	**34,068**	**33,654**	**27,474**	0,316	5,195	1,612	3,183	**BCLAF1**	**-15,679**	1,642	0,000078	**BCLAF1**	**-15,266**	5,204	0,007077	**BCLAF1**	**-6,181**	3,5682	0,039932	same
**MLH3**	**71,939**	**73,898**	**53,798**	**71,214**	2,661	3,761	6,207	2,981	**MLH3**	**-18,142**	6,754	0,009643	**MLH3**	**1,958**	4,607	0,502464	**MLH3**	**17,416**	6,886	0,011868	co-opt
**SDCCAG8**	**80,291**	**77,694**	**59,167**	**58,575**	3,116	2,721	1,975	3,038	**SDCCAG8**	**-21,124**	3,690	0,000581	**SDCCAG8**	**-2,597**	4,137	0,337997	**SDCCAG8**	**-0,592**	3,6239	0,791392	
**CASP8**	**54,469**	**57,051**	**30,998**	**36,249**	1,126	0,452	0,518	2,244	**CASP8**	**-23,472**	1,240	0,000005	**CASP8**	**2,582**	1,214	0,021123	**CASP8**	**5,251**	2,3033	0,016835	co-opt
**RBBP8**	**68,838**	**75,950**	**44,809**	**62,272**	1,439	1,173	1,928	1,295	**RBBP8**	**-24,029**	2,406	0,000066	**RBBP8**	**7,112**	1,857	0,002680	**RBBP8**	**17,463**	2,3227	0,000201	same
**BRCA1**	**80,373**	**80,928**	**41,445**	**60,067**	3,713	1,919	4,383	3,242	**BRCA1**	**-38,928**	5,744	0,000301	**BRCA1**	**0,556**	4,180	0,829185	**BRCA1**	**18,622**	5,4518	0,004087	co-opt
**BCL2L1**	**89,448**	**94,216**	**48,351**	**64,287**	2,149	1,240	7,322	3,011	**BCL2L1**	**-41,097**	7,631	0,000735	**BCL2L1**	**4,768**	2,481	0,029128	**BCL2L1**	**15,936**	7,9167	0,025201	co-opt
**CHEK2**	**97,126**	**96,219**	**54,105**	**67,777**	0,399	1,022	4,920	1,694	**CHEK2**	**-43,021**	4,936	0,000112	**CHEK2**	**-0,908**	1,097	0,225205	**CHEK2**	**13,672**	5,2031	0,010409	co-opt
**DOM3Z**	**82,989**	**80,912**	**20,822**	**27,076**	1,106	2,483	1,288	2,512	**DOM3Z**	**-62,167**	1,697	0,000000	**DOM3Z**	**-2,077**	2,718	0,256214	**DOM3Z**	**6,254**	2,823	0,018501	co-opt

SUPPLEMENTARY TABLE S5

IDENTIFICATION			BIOINFORMATIC PREDICTION OF THE PRESENCE OF RNA BINDING PROTEINS MOTIFS										ENCODE eCLIP DATA		
			Motifs Analysis and Prediction of RNA Binding Proteins (rbpmap.technion.ac.il/)* (High stringency)						RBPDB: The database of RNA-binding specificities (rbpdb.ccbr.utoronto.ca)** (Threshold 0.75)				(https://www.encodeproject.org/)*** Number of signal peaks; intensity scale (1 to 6)		
SPLICING UNIT	ALTERNATIVE and FLANKING SEQUENCES		HNRNP-A1		KHDRBS1 (Sam68)		SRSF10		HNRNP-A1		SRSF10		HNRNP-A1		KHDRBS1(Sam68)
Name	Size bp	Genomic Coordinates	Matching sequence	Position	Matching sequence	Position	Matching sequence	Position	Matching sequence	Position	Matching sequence	Position	HepG2 cells	K562 cells	K562 cells
**BCL2L1**	860	chr20:31721353-31722212	uugggga	245			agagaua	224			AAAGGCC	438	5; 6	3; 6	6; 2-3-6-6-3-2
							aaagaaa	268			AAAGAAA	268			
							agagaaa	827			AAAGAGA	222			
											AAAGGGA	273			
											AAAGAUG	97			
											AGAGGCC	261			
											AGAGAAA	827			
											CAAGGAG	138			
											CAAGGGG	284			
**TNFSRF10**	871	chr8:23028027-23028898	auagggu	244			agagacc	123	UAGGGU	245	AAAGAGG	637	4;5	3;2	4;2-2-2-1
							agagaga	419			AAAGACU	349			
							agagaac	421			AGAGAAC	421			
											AGAGACC	123			
											AGAGGAG	639			
											AGAGGGG	597			
											AGAGAGU	266			
											AGAGAGA	419			
											AGAGGGA	591			
											AGAGGGA	112			
**BRCA1**	581	chr17:43095595-43096176	aguuag	448	auaagaa	353	agagaau	113	UAGGGA	332	AAAGGGC	23	0;0	0;0	3;1-1-3
									UAGGUA	57	AAAGGUC	156			
											AAAGACA	338			
											AGAGAAU	113			
	535	chr17:43096997-43097532	augaaaa	68			ggagaaa	10	UAGGAA	204	AAAGGGU	343	0;0	0;0	1;1
			auaaaag	147			aaagaaa	22			AAAGAAA	22			
			uuaaaau	154			agagaga	171			AAAGAGA	169			
			uuaaaaa	165			agagaaa	173			AAAGGCA	461			
			cuaaaaa	294			agagaag	449			AAAGGUU	228			
			auaaaag	324							AGAGAAG	449			
			auuaaaa	339							AGAGAAA	173			
			uuaaaag	340							AGAGAGA	171			
			uuaaaaa	349							AGAGGGA	6			
**CASPASE 8**	703	chr2:201271217-201271920	gguuag	446	aaaaaag	28	agaaaaa	21			AAAGACU	669	3;1	0;0	3;2-1-1
					agaaaag	69	aaagaaa	67			AAAGAAA	67			
							agagagg	475			AAAGGAA	31			
											AAAGAGA	473			
											AGAGGCC	615			
											AAAGAUU	265			
											AGAGAGG	475			
											CAAGGGC	157			
											AGAGGGG	477			
											AGAGGAA	369			
	738	chr2:201272341-201273079			auuaaaa	223	ggagaaa	210			AAAGAGU	368	1;2	0;0	3;1
					uuaaaaa	224	agagaga	556			AGAGACC	157			
					cuaaaaa	243	agagaag	558			AGAGAAG	558			
											CAAGAGC	390			
											CAAGGGC	191			
											AGAGGGU	329			
											AAAGGUA	433			
											AGAGAGA	556			
	658	chr2:201274581-201275239	ggaggagu	310	uuaaaaa	209	agagaac	350			AAAGACC	214	1;1	0;0	3;1
			ggaguugu	313	aaaaaag	211	agaaaaa	505			AGAGAAC	350			
					aaaaaag	521					AAAGUGC	157			
					uuaaaaa	528					CAAGGGG	468			
**CASPASE 9**	739	chr1:15504318-15505057	guuggagu	478	augaaaa	311	ggagaaa	225	UAGGAA	485	AAAGAGC	316	2;2-1	3;3-1-1	3;1-2-2
											AAAGACC	587			
											AAAGAAG	229			
											AAAGGGA	158			
											AGAGACC	193			
											AAAGACA	609			
											CAAGAAC	451			
											CAAGAAG	472			
											AGAGGGA	269			
											CAAGGGG	625			
	695	chr1:15505679-15506374			auaaaag	472	agagaga	324			AAAGGGC	475	2;1	1;1	2;1-2
					cuaaaaa	607	agagaau	326			AGAGGAC	143			
											AGAGGGC	89			
											AGAGGGC	409			
											AGAGAGG	407			
											AGAGAAU	326			
											CAAGAAG	229			
											AGAGAGA	324			
	770	chr1:15506596-15507366	agauag	289			agagagc	113			AAAGGAC	727	2;2	5;1	3;1-3-1
			gcagaagu	418			agagaag	618			AGAGGAC	529			
			guagcagg	584							AGAGAGC	113			
			uaaggga	674							AAAGAGA	616			
											AGAGAAG	618			
											AAAGAUA	244			
											AGAGGCG	543			
											CAAGGAG	40			
											CAAGGAG	578			
	636	chr1:15507573-15508209	cuagggu	191			agagaaa	346	UAGGGU	192	AAAGAGC	554	2;1	4;2-2-1-1	4;1-3-1-1
							agagaga	352	UAGGUA	542	AAAGACU	581			
							agagaaa	354			AAAGAGA	350			
											CAAGAAC	417			
											CAAGAGC	327			
											AGAGGAG	381			
											AGAGAGU	28			
											AGAGAAA	354			
											AGAGAAA	346			
											AGAGAGA	352			
											CAAGGAG	560			
											CAAGGGG	391			
**CHEK2**	663	chr22:28703199-28703862			agaaaag	16	ggagaaa	381			AAAGGGA	388	0;0	2;1	3;1-2-2
					uuaaaaa	287	agaaaaa	383			AAAGGCA	223			
					auaauaa	321	ugagaaa	401			CAAGAAC	464			
					aucaaaa	336					CAAGAGC	426			
					aaaaaag	339					AGAGGGG	375			
					aaaaaag	385					AGAGACU	589			
											AGAGGCU	492			
											AGAGGAA	414			
											AGAGGGA	448			
											CAAGAGG	412			
											CAAGGAU	613			
**DOM3Z**	481	[illegible]_GL000254v2_alt:3312667-331314[illegible]	gcagcagg	215			agagaag	206			AGAGAAG	206	0;0	0;0	0;0
			gcaggcgu	218							AGAGGGG	167			
											AGAGGGU	102			
											CAAGGAG	201			
**RBPP8**	586	chr18:22933874-22934460	guaguggu	221	auuaaac	558	agagaga	356			AAAGGAU	496	0;0	0;0	0;0
							agagaga	358			AAAGGGU	400			
											AGAGGAC	96			
											AAAGUGC	374			
											AGAGAGA	356			
											AGAGAGA	358			
**SPTAN1**	658	chr9:128592681-128593339	gaagaggu	514							AAAGAUG	458	5;2-2-2-1-1	0;0	1;1
			gcagcugu	528							AGAGGUC	516			
			gcagcugu	576							CAAGGGA	509			
			agaguggu	590											

* Paz I, Kosti I, Ares M Jr, Cline M, Mandel-Gutfreund Y. RBPmap: a web server for mapping binding sites of RNA-binding proteins. Nucleic Acids Res., 2014, PMID: 24829458
** Cook KB, Kazan H, Zuberi K, Morris Q, Hughes TR. Nucleic Acids Research, Volume 39, Issue suppl_1, 1 January 2011, Pages D301–D308, PMID: 21036867
*** Van Nostrand EL, Pratt GA, Shishkin AA, Gelboin-Burkhart C, Fang MY, Sundararaman B, Blue SM, Nguyen TB, Surka C, Elkins K, Stanton R, Rigo F, Guttman M, Yeo GW.
Robust transcriptome-wide discovery of RNA-binding protein binding sites with enhanced CLIP (eCLIP). Nat Methods. 2016 Jun;13(6):508-14. doi: 10.1038/nmeth.3810. Epub 2016 Mar 28.PMID: 27018577

SUPPLEMENTARY TABLE S6

Gene	Unit Name	Size of PCR products		Alternative event		Primer Name		Primer Sequence	
		S (bp)	L (bp)	size (bp)	type	Forward	Reverse	Forward	Reverse
**AKIP1**	C11orf17-F1 C11orf17-R1	252	333	81	exon cassette	C11orf17-F1	C11orf17-R1	CTCTAGAAGTGCTGGAGAG	GACCATTCCCTATGTCCAAG
**APAF1**	APAF1-3 APAF1-4	768	897	129	exon cassette	APAF1-3	APAF1-4	GGACCCTCAAGAGGATATG	GAAAGTACTGTACCCTGGTG
**ARID4B**	RBP1L1-F1 RBP1L1-R1	625	883	258	exon cassette	RBP1L1-F1	RBP1L1-R1	GAGGAGTACTGTAGATCAGC	TGGCATCAGTGAGATCCAG
**AURKA**	rs.AURKA.F1 rs.AURKA.R1	119	217	98	exon cassette	rs.AURKA.F1	rs.AURKA.R1	CCAGAGTGCGGGGATATTTGAT	TGCAGTTTTCTTTAGATCGGTCCA
**AURKB**	AURKB.ec2.F1 AURKB_G_r	199	272	73	exon cassette	AURKB.ec2.F1	AURKB_G_r	CGGGGCGGGAGATTTGAAAAGT	TGTGGGCTGGACATTGGAGC
**AXIN1**	AXIN1-3 AXIN1-4	535	643	108	exon cassette	AXIN1-3	AXIN1-4	GAGGCTACTCAGAGAGTGTT	CAGAAGTAGTACGCCACAAC
**AXL**	primer-1291 primer-1292	373	400	27	exon cassette	primer-1291	primer-1292	CCCCTGAGAACATTAGTGCT	AGAGCCAAGATGAGGACACA
**BCL2L1**	BCLX-3 BCLX-2	267	456	189	alternative 5'ss	BCLX-3	BCLX-2	ATGGCAGCAGTAAAGCAAGCG	TCATTTCCGACTGAAGAGTGA
**BCL2L11**	113317 BCL2L11-4	130	220	90	exon cassette	113317	BCL2L11-4	TACCTCCCTACAGACAGAG	CCTCCTTGCATAGTAAGCGT
**BCLAF1**	BCLAF1.u.f.6 BCLAF1.u.r.6	160	679	519	alternative 3'ss	BCLAF1.u.f.6	BCLAF1.u.r.6	CACCACAGAATGCTCCAAGA	GGGCTTTCCTCTCTGAAGGT
**BCLAF1**	NM_014739-3 NM_014739-4	479	626	147	exon cassette	NM_014739-3	NM_014739-4	GTACCCTGAGGAAGCATAC	AGTACCACGACCTCTTCCT
**BNIP1**	BNIP1-5 BNIP1-6	339	468	129	exon cassette	BNIP1-5	BNIP1-6	CCGGATCTGTAACCAAGAG	CTTAAGAGATCTCCTCCCTG
**BRCA1**	BRCA1-F2 BRCA1-R2	287	410	123	double exon cassette	BRCA1-F2	BRCA1-R2	GTCTCAGTGTCCAACTCTCT	GTCACTCTGAGAGGATAGC
**GPR137**	C11ORF4.F3 C11ORF4.R3	124	274	150	exon cassette	C11ORF4.F3	C11ORF4.R3	ACTCCCTGTTCGTCATCTGC	TTGTTCCCCAGGTCATTCAC
**CASC4**	NM_177974-F1 NM_177974-R1	241	409	168	exon cassette	NM_177974-F1	NM_177974-R1	CAACTGGACAACCTCTCTC	GCAGGATCCATTTGAAGCTC
**CASP8**	CASP8-3 CASP8-4	413	433	20	reciprocal exon cassette	CASP8-3	CASP8-4	CACTAGAAAGGAGGAGATGG	GATGATCAGACAGTATCCCC
**CASP9**	primer-113212 primer-113213	360	670	310	alt. 5'ss +multiple exon cassette	primer-113212	primer-113213	TCCTGCTTAGAGGACACAG	GGACACAAAGATGTCACTGG
**CDC25A**	refseq_CDC25A.F1 refseq_CDC25A.R1	145	265	120	exon cassette	refseq_CDC25A.F1	refseq_CDC25A.R1	CGACCCAGATGAGAACAAGG	ATCGAGAAGGTCCACGAAGC
**CDC25B**	refseq_CDC25B.F3 refseq_CDC25B.R3	101	224	123	exon cassette	refseq_CDC25B.F3	refseq_CDC25B.R3	GAGCAGTTTGCCATCAGACG	TCTGCCAGAGCATGGGTG
**CEP170**	rs.CEP170.F1 rs.CEP170.R1	250	358	108	alternative 3'ss	rs.CEP170.F1	rs.CEP170.R1	CGCAGAAGCCATCATTAGAAGTG	TCGATGAGCAGTCCAATCTCGG
**CHEK1**	CHEK1.e.F1 CHEK1.e.R1	103	327	224	exon cassette	CHEK1.e.F1	CHEK1.e.R1	GACTGGGACTTGGTGCAAAC	TGCCATGAGTTGATGGAAGA
**CHEK2**	CHEK2.u.f.31 CHEK2.u.r.29	194	256	62	exon cassette	CHEK2.u.f.31	CHEK2.u.r.29	CAGCTCTCAATGTTGAAACAGAA	TCTGGCTTTAAGTCACGGTGT
**GTF2H2**	refseq_DKFZP686M0199.F1 refseq_DKFZP686	152	250	98	exon cassette	refseq_DKFZP686M0199.F1	refseq_DKFZP686M0199.R1	GTTGAATTGCCCTGCCTG	GTTCTTTCATAGCCTCCTTCCC
**DOM3Z**	DOM3Z.e1.F1 DOM3Z.e1.R1	241	314	73	alternative 3'ss	DOM3Z.e1.F1	DOM3Z.e1.R1	ATAGTGACGTGGCGGGGGCA	GAGAAGAGCAGAGGGTGGCTTC
**ESYT2**	FAM62B.U.1.F1 FAM62B.U.1.R1	124	187	63	exon cassette	FAM62B.U.1.F1	FAM62B.U.1.R1	TCAAAGCTGACAAAGACCAAGC	TGGACAACAGGATTTGGGTTGC
**EXO1**	EXO1.ec1.F1 EXO1.ec1.R1	193	298	105	exon cassette	EXO1.ec1.F1	EXO1.ec1.R1	ACTATCGCACTCAGCCATTCTT	TGTAGCAATCCCTGTATCCCCA
**F3**	F3.F2 F3.R2	270	430	160	exon cassette	F3.F2	F3.R2	CTCGGACAGCCAACAATTCA	CCACTCCTGCCTTTCTACACTT
**FANCA**	FANCA.F13 FANCA.R13	102	227	125	exon cassette	FANCA.F13	FANCA.R13	AACCTGAAGCTGATGCTCTTTC	TATCCTCATTTCCTGTGCGG
**FASTK**	FAST_HUMAN-5 FAST_HUMAN-6	410	730	320	double exon cassette	FAST_HUMAN-5	FAST_HUMAN-6	CTGCTCTCTGCTCAGACCT	GGACCAACTTCTGTACCAC
**RP11-517H2.6**	FGFR1OP.u.f.10 FGFR1OP.u.r.12	108	168	60	exon cassette	FGFR1OP.u.f.10	FGFR1OP.u.r.12	CCAAAGTCACCAGAGGGAAA	GCTCTTGGGTTCTGACAAGG
**FOXM1**	refseq_FOXM1.F2 refseq_FOXM1.R2	100	145	45	exon cassette	refseq_FOXM1.F2	refseq_FOXM1.R2	CAGTGCCAACCGCTACTTGA	GGGGAGTTCGGTTTTGATG
**INCENP**	rs.INCENP.F1 rs.INCENP.R1	107	119	12	exon cassette	rs.INCENP.F1	rs.INCENP.R1	CCGACCTCAGCCCCACGCAG	CGCCGCAGATTCTCCAGGCG
**KIF13A**	rs.KIF13A.F1 rs.KIF13A.R1	134	173	39	exon cassette	rs.KIF13A.F1	rs.KIF13A.R1	TGCCACTTATGGTTGAAGCCA	TGCATCTGACCACCTCTCCCTT
**KITLG**	KITLG.u.f.9 KITLG.u.r.10	215	299	84	exon cassette	KITLG.u.f.9	KITLG.u.r.10	TGATGCCTTCAAGGACTTTGT	CTGCCCTTGTAAGACTTGGC
**LGALS9**	LGALS9.F10 LGALS9.R6	135	231	96	exon cassette	LGALS9.F10	LGALS9.R6	GTGATGGTGAACGGGATCCT	GTTGGCAGGCCACACGCC
**LIG3**	LIG3.u.f.1 LIG3.u.r.6	353	378	25	alternative 5'ss	LIG3.u.f.1	LIG3.u.r.6	CGGATTTAAAGAGACAGGCG	GTTGCTCAGCCATCTCACAG
**MAP2**	refseq_MAP2.F3 refseq_MAP2.R3	300	393	93	exon cassette	refseq_MAP2.F3	refseq_MAP2.R3	CAAGTTATTCTTCACGCACACC	TGGATGTCACATGGCTTAGG
**MAPK10**	refseq_MAPK10.F1 refseq_MAPK10.R1	138	197	59	exon cassette	refseq_MAPK10.F1	refseq_MAPK10.R1	CAGAACCAAACACATGCCAA	CGCTCATGGTCTAATTCCATCT
**MCL1**	MCL1.F1 MCL1.R1	134	382	248	exon cassette	MCL1.F1	MCL1.R1	CCAAGGACACAAAGCCAATG	TGGAAGAACTCCACAAACCC
**MLH3**	refseq_MLH3.F1 refseq_MLH3.R1	153	225	72	exon cassette	refseq_MLH3.F1	refseq_MLH3.R1	CCCTATCGTTTCACCAAAGG	TTTCCGACCAGAGCCTTGT
**NAP1L4**	NAP1L4.e1.F2 NAP1L4.e1.R2	341	379	38	exon cassette	NAP1L4.e1.F2	NAP1L4.e1.R2	CGAGGAGGGAGAAGACGAGGAT	GAAGTCCAGAGCTACAGGCAC
**NDEL1**	refseq_NDEL1.F2 refseq_NDEL1.R2	130	165	35	exon cassette	refseq_NDEL1.F2	refseq_NDEL1.R2	ACCAAGCATCACGAAAATCC	GGGTCAAAGCCGTTTACTGC
**PCBP4**	PCBP4.ec1.F1 PCBP4.ec1.R1	188	317	129	exon cassette	PCBP4.ec1.F1	PCBP4.ec1.R1	GCAAATGGTGGAAATGTCTCCAG	TGGTTGGCAGAGAGAAGAACAG
**POGZ**	refseq_POGZ.F1 refseq_POGZ.R1	200	359	159	exon cassette	refseq_POGZ.F1	refseq_POGZ.R1	ACCTGTTCATGGAATGTGAGG	TGGTTGAGTAACCATTGTGCC
**PPP3CB**	PPP3CB.u.f.21 PPP3CB.u.r.22	117	147	30	exon cassette	PPP3CB.u.f.21	PPP3CB.u.r.22	ACAGGGATGTTGCCTAGTGG	ATCCAAACCCTTTGCCTCTT
**RBBP8**	refseq_RBBP8.F1 refseq_RBBP8.R1	145	226	81	alternative 5'ss	refseq_RBBP8.F1	refseq_RBBP8.R1	AATGTGCCTCTCGCCTTACC	TGCTCCACACTTCTACTTGCTT
**RCC1**	rs.SNHG3-RCC1.F1 rs.SNHG3-RCC1.R1	103	246	143	exon cassette	rs.SNHG3-RCC1.F1	rs.SNHG3-RCC1.R1	AGGGTCCTTTATATAGAAGGGAGAGT	CTTCTTGCTTTTGGGGATGGCA
**SDCCAG8**	SDCCAG8-F1 SDCCAG8-R1	180	417	237	double exon cassette	SDCCAG8-F1	SDCCAG8-R1	GTGCCTGAGACTAACAGAAC	ATCGTCTCATGTACTCTCCC
**SLIT2**	SLIT2.F36 SLIT2.R4	222	234	12	exon cassette	SLIT2.F36	SLIT2.R4	GGCAAGTTTCAACCATATGCC	GGAGCCATAAATGACTGGTGAC
**SPTAN1**	SPTAN1.ec1.F1 SPTAN1.ec1.R1	308	368	60	exon cassette	SPTAN1.ec1.F1	SPTAN1.ec1.R1	GGTGGAAAGTGGAAGTGAACGA	AACCTGCTCCAAGTCTGCTCC
**TNFRSF10B**	TNFRSF10B-3 TNFRSF10B-4	584	660	76	intron retention	TNFRSF10B-3	TNFRSF10B-4	CAGGACTATAGCACTCACTG	CTCCTCCTCTGAGACCTTT
**TSSC4**	TSSC4.F10 TSSC4.R7	140	332	192	intron retention	TSSC4.F10	TSSC4.R7	TTGGCTGTCCAATCACACTC	ATGCCTCTCAGATGGAATGG
**UTRN**	UTRN.u.f.81 UTRN.u.r.78	231	270	39	exon cassette	UTRN.u.f.81	UTRN.u.r.78	CAAACACCCTCGACTTGGTT	TGGCAATACTGCTGGATGAG
**ZWINT**	refseq_ZWINT.F2 refseq_ZWINT.R2	234	375	141	alternative 5'ss	refseq_ZWINT.F2	refseq_ZWINT.R2	GGGAAGCCTTTGAGCAGC	TCCCCATGGTGTCTCCTG
